# Effects of Aneuploidy on Genome Structure, Expression, and Interphase Organization in *Arabidopsis thaliana*

**DOI:** 10.1371/journal.pgen.1000226

**Published:** 2008-10-17

**Authors:** Bruno Huettel, David P. Kreil, Marjori Matzke, Antonius J. M. Matzke

**Affiliations:** 1 Gregor Mendel Institute of Molecular Plant Biology, Austrian Academy of Sciences, Vienna, Austria; 2 Chair of Bioinformatics, Boku University Vienna, Vienna, Austria; The Salk Institute for Biological Studies, United States of America

## Abstract

Aneuploidy refers to losses and/or gains of individual chromosomes from the normal chromosome set. The resulting gene dosage imbalance has a noticeable affect on the phenotype, as illustrated by aneuploid syndromes, including Down syndrome in humans, and by human solid tumor cells, which are highly aneuploid. Although the phenotypic manifestations of aneuploidy are usually apparent, information about the underlying alterations in structure, expression, and interphase organization of unbalanced chromosome sets is still sparse. Plants generally tolerate aneuploidy better than animals, and, through colchicine treatment and breeding strategies, it is possible to obtain inbred sibling plants with different numbers of chromosomes. This possibility, combined with the genetic and genomics tools available for *Arabidopsis thaliana*, provides a powerful means to assess systematically the molecular and cytological consequences of aberrant numbers of specific chromosomes. Here, we report on the generation of *Arabidopsis* plants in which chromosome 5 is present in triplicate. We compare the global transcript profiles of normal diploids and chromosome 5 trisomics, and assess genome integrity using array comparative genome hybridization. We use live cell imaging to determine the interphase 3D arrangement of transgene-encoded fluorescent tags on chromosome 5 in trisomic and triploid plants. The results indicate that trisomy 5 disrupts gene expression throughout the genome and supports the production and/or retention of truncated copies of chromosome 5. Although trisomy 5 does not grossly distort the interphase arrangement of fluorescent-tagged sites on chromosome 5, it may somewhat enhance associations between transgene alleles. Our analysis reveals the complex genomic changes that can occur in aneuploids and underscores the importance of using multiple experimental approaches to investigate how chromosome numerical changes condition abnormal phenotypes and progressive genome instability.

## Author Summary

Most plants and animals have two copies of each chromosome in the normal chromosome set. Unbalanced numerical changes resulting from gains or losses of individual chromosomes (aneuploidy) usually have deleterious consequences. For example, Down syndrome in humans is caused by an extra (triplicate) copy of chromosome 21. Human tumor cells usually display numerous alterations in chromosome number and structure. Little is known about how changes in chromosome number influence gene activity and chromosome integrity, thereby perturbing physiology and development. We have used the model plant *A. thaliana* to study how triplication of chromosome 5 affects gene expression, chromosome structure, and chromosome packaging in the nucleus. The results indicate that the presence of an extra chromosome 5 has multiple effects: (1) substantial changes in gene expression occur, primarily on the triplicated chromosome 5 but also on the four non-triplicated chromosomes; (2) broken derivatives of chromosome 5 can be retained in the presence of two normal copies; and (3) two copies of the triplicated chromosome 5 may show a slightly enhanced tendency to associate with each other, perhaps to spatially compensate for the chromosome imbalance. The detrimental effects of aneuploidy are likely due to concurrent changes in gene expression, chromosome structure, and arrangement.

## Introduction

Changes in the number of chromosomes from the normal diploid set can be grouped into two types: polyploidy and aneuploidy. Polyploidy refers to whole genome duplications whereas aneuploidy refers to unbalanced losses and/or gains of individual chromosomes, or parts of chromosomes, from the basic chromosome set. Early work on plants and insects revealed that aneuploidy has a greater effect on phenotype than polyploidy [1],[2]. These observations can be explained in terms of the gene balance hypothesis, which posits that dosage imbalances of genes encoding regulatory molecules disturb their stoichiometry within multi-protein complexes and disrupt cellular processes [2]. Consistent with this hypothesis, work in *Drosophila* has indicated that genes encoding transcription factors and members of signal transduction cascades are primarily responsible for dosage effects on the phenotype [1].

The gene balance hypothesis provides a conceptual framework for investigating in greater detail the molecular and cytological consequences of aneuploidy. This information is important for understanding the coordinated operation and expression of the genome as well as syndromes and disease states associated with abnormal chromosome numbers. The latter is exemplified by human solid tumour cells, which are highly aneuploid. The karyotypes of advanced tumour cells typically feature not only a plethora of chromosome numerical aberrations but also extensive structural alterations, including translocations and deletions [3]. The co-existence of chromosome numerical and structural changes in tumour cell nuclei hints that they are linked in some way, but the basis of this connection is unclear. The genomes of tumour cells often display a distinctive DNA methylation profile that is characterized by global hypomethylation accompanied by aberrant hypermethylation of CpG islands within promoter regions [4],[5]. That aneuploidy might be at the root of these diverse genomic and epigenomic changes was suggested by a study on trisomic tobacco plants, in which the chromosome present in triplicate was prone to breakage, local increases in DNA methylation, and gene silencing [6],[7].

Another aspect of aneuploidy concerns interphase chromosome arrangement and dynamics, which are increasingly regarded as factors influencing gene activity [8]. Down syndrome in humans, which is caused by triplication of chromosome 21 (trisomy 21), is relevant in this context. Chromosome 21 is the smallest human autosome [9], not the most gene-poor (a distinction that belongs to chromosome 13 [10]), and it is the only autosome that is compatible with extended life after birth when triplicated [11]. These observations might be partially explained if extra chromosomes interfere with chromosome packaging or mechanics such that triplication of the smallest is the least harmful. However, the ways in which extra or missing chromosomes in aneuploids might perturb the three-dimensional (3D) architecture and dynamics of interphase chromosomes are not understood.

The consequences of aneuploidy for global gene expression patterns are only beginning to be assessed. With respect to Down syndrome, the naïve expectation is that genes on the triplicated chromosome 21 will be expressed at 1.5 times the level found in chromosome 21 disomics according to the increase in gene dosage. However, only a subset of expressed genes on triplicated chromosome 21 appears to be up-regulated in the expected manner whereas the expression of many genes is adjusted to the disomic level, indicating dosage compensation [12]. The extent of trans or secondary effects, in which genes on non-triplicated chromosomes are misregulated, is still not fully resolved with respect to trisomy 21 [13]–[15]. Trans effects have been documented in aneuploids of maize [16],[17] and yeast [18], demonstrating that changes in expression are not restricted to genes on the numerically altered chromosome. However, information about how global patterns of gene expression are adjusted following chromosome-wide alterations in gene dosages is still limited. This issue is complex because unique expression profiles are likely to result from numerical changes of specific chromosomes or chromosome regions.

Plants have traditionally provided excellent systems for studying aneuploidy. The terms trisome and monosome were coined by Blakeslee, Belling and coworkers from their classic work in the 1920's on the twelve trisomics of *Datura stramonium* (Jimson weed), each of which displays a distinctive phenotype [2]. With respect to mechanisms of epigenetic regulation and genome composition, plants are arguably more similar to mammals than are yeasts or *Drosophila*. For example, both plants and mammals have DNA methylation, histone H3 lysine 9 and lysine 27 methylation, and proteins of the RNAi machinery; moreover, their genomes contain substantial amounts of repetitive DNA, which can potentially affect gene expression and chromosome structural stability [19]. Insights gained from plants can thus be informative for understanding the effects of aneuploidy in mammalian cells. Plants have the advantage of generally tolerating aneuploidy better than mammals, and their chromosome numbers can be more easily manipulated to allow systematic analyses of the consequences of chromosome numerical aberrations.

We are using the model plant *Arabidopsis thaliana* (2n = 10) to investigate the impact of aneuploidy on genome structure, expression and 3D organization of interphase chromosomes. All five trisomics of *Arabidopsis* (2n = 10+1) are viable and have a distinctive phenotype [20]. The genetics and genomics resources available for *Arabidopsis* are unsurpassed in the plant kingdom. In addition, transgenic *Arabidopsis* lines are available in which distinct chromosome sites are tagged with fluorescent markers [21],[22], allowing the identification of specific trisomics at an early stage and subsequent live cell imaging of fluorescent-tagged sites in interphase nuclei in intact plants. Here we report the results of experiments using these tools to analyze the molecular and cytological consequences of chromosome 5 triplication in *Arabidopsis*.

## Results/Discussion

### Identification of Chromosome 5 Trisomic Plants in F2 and F3 Generations

**Figure 1:**
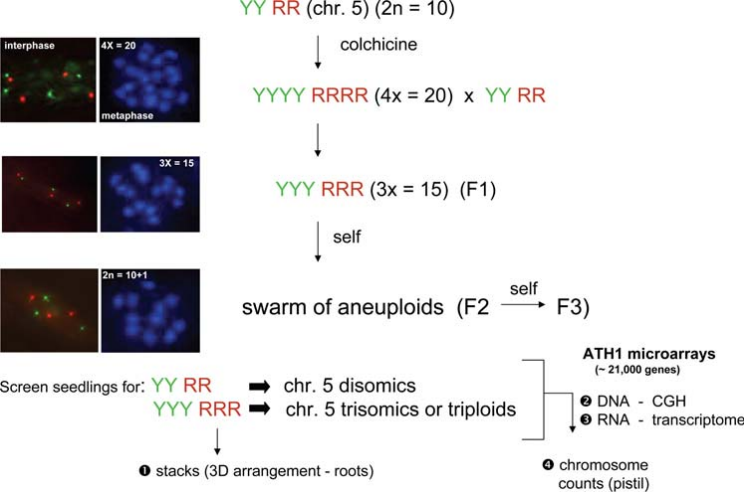
Experimental strategy. We started with a normal diploid plant that was doubly homozygous for two fluorescent-tagged sites on chromosome 5: YFP (Y) on the top arm and DsRed (R) on the bottom arm (Figure 2A). Diploid seedlings (2Y 2R) were treated with colchicine to produce tetraploids (4Y 4R). Crosses between a tetraploid and diploid produced triploid progeny (3Y 3R) (F1 generation). Self-fertilization of a triploid produces a ‘swarm of aneuploids’ [47], including various trisomics [48]. At the seedling stage, progeny of the triploids (F2 generation) were examined under a fluorescence microscope to determine the number of fluorescent signals in interphase nuclei of roots, which have a low background fluorescence at the excitation wavelengths for both YFP and DsRed. Three DsRed dots and three YFP dots (3R 3Y) identified seedlings that were either chromosome 5 trisomics or triploids. Optical sections were made from root nuclei in living seedlings to obtain stacks for 3D reconstructions of interphase nuclei from chromosome 5 trisomics and from triploids. Seedlings were then planted in soil and DNA and RNA were isolated from rosette leaves. DNA was used for array CGH to detect chromosome numerical imbalances and the approximate locations of chromosome breaks; RNA was used for transcript profiling. The plants were allowed to flower and metaphase chromosome counts were performed using pistil material. F3 progeny were obtained by self-fertilization of F2 plants.

**Figure 2:**
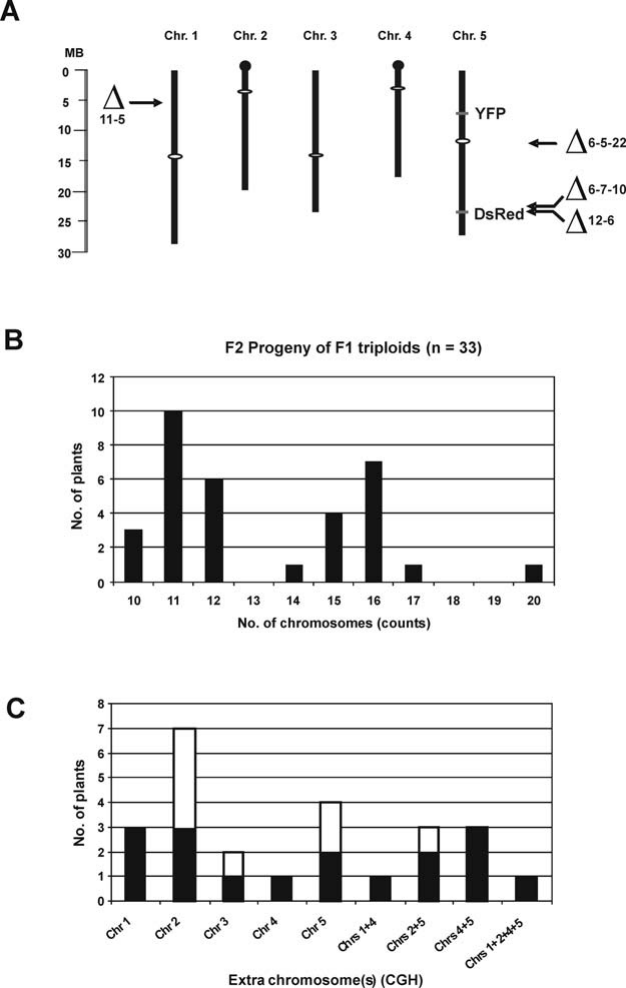
Chromosomal positions of deletions and transgenes, and chromosome constitution of aneuploids. A: *Arabidopsis* chromosomes showing approximate sizes in megabases (MB), positions of centromeres (white ovals), nucleolar organizers (black balls), and *YFP* and *DsRed* transgene inserts on chromosome 5, as well as the approximate chromosome breakpoints detected by array comparative genome hybridization (CGH) in the indicated chromosome 5 trisomic (6-5-22, 6-7-10 and 12-6) and triploid (11-5) plants. The positions of the breakpoints are estimated to be around the last gene that yields a trisomic signal. The breakpoint in plant 11-5 is around At1g15660 located at 5.38 MB on the top arm of chromosome 1; in plant 6-5-22 it is around At5g32440, which is in the pericentromeric heterochromatin on the bottom arm of chromosome 5; in plant 6-7-10, it is around At5g58040; and in plant 12-6 it is close to the *Arabidopsis* DNA and transgene DNA junction at around At5g58140. B: Array CGH identified chromosome imbalances in 33 F2 progeny obtained from self-fertilization of F1 triploids and metaphase chromosome counts determined the chromosome number (Table S1A). Trisomics (2n = 10+1) were the most common unbalanced karyotype in F2 progeny. Balanced diploids (2n = 10), triploids (3X = 15) and tetraploids (4X = 20) were also obtained. The distribution is similar to one described previously [49]. C: Distribution of extra chromosomes in unbalanced karyotypes. All 5 *Arabidopsis* chromosomes were detectable as simple aneuploids (one chromosome numerically altered), while only a subset of combinations was observed in ‘extreme’ aneuploids (more than one chromosome numerically altered). Black areas in columns show the number of plants with extra chromosomes in a diploid background; white areas show the number of plants with extra chromosomes in a triploid background.

The strategy for obtaining chromosome 5 trisomics and for subsequent analysis of these plants is shown in Figure 1. We started with a diploid parental line that was homozygous for DsRed (R) and YFP (Y) fluorescent tags on chromosome 5, which is one of the largest chromosomes in *Arabidopsis* (Figure 2A). From a cross between the diploid parent and a tetraploid derivative produced by colchicine treatment, we obtained triploid plants (F1 generation). Self-fertilization of F1 triploids produced F2 progeny, 33 of which were selected for more detailed investigation. Screening root nuclei in F2 seedlings for chromosome 5 fluorescent tags allowed us to predict whether individual F2 plants might be diploid (2R 2Y), chromosome 5 trisomic/triploid (3R 3Y) or chromosome 5 tetrasomic/tetraploid (4R 4Y). The actual chromosome numbers were subsequently determined by counting metaphase chromosomes, and the presence of unbalanced chromosome sets was assessed by array comparative genome hybridization (CGH) (Table S1).

The F2 progeny comprised a complex population containing chromosomally balanced diploids, triploids and tetraploids, as well as chromosomally unbalanced trisomics (the most frequently observed chromosome constitution), double trisomics (2n = 10+1+1), and near triploids (3X = 15+/−1 or 15+1+1) (Figure 2B). As expected from the screen of chromosome 5 fluorescent tags, we obtained a number of plants with a triplicated chromosome 5 (3R 3Y); however, subsequent array CGH and metaphase chromosome counts revealed that only three of these were true triploids (plants 8-5, 8-6, 9-1; plant 11-5 had 15 chromosomes, but one copy of chromosome 1 was truncated; see below) and just two were simple chromosome 5 trisomics (plants 6-5 and 6-7) (Table S1A). The remaining ‘3R 3Y’ plants had an additional extra chromosome(s), the most common being either chromosome 2 or 4, which are the smallest of the *Arabidopsis* chromosome set (Figure 2C).

Representatives of the next generation (F3) were obtained by self-fertilization of the two trisomic F2 plants (6-5 and 6-7) and two diploid F2 siblings (6-4 and 7-2). From each of the two trisomic F2 parents, we selected around a dozen F3 progeny that were identified by fluorescence microscopy as potential chromosome 5 trisomics (3R 3Y) (Table S1B). Extra copies of chromosome 5 were confirmed in these plants by array CGH and, in most cases, the expected chromosome number (2n = 10+1) was established by counting metaphase chromosomes. From each of the two diploid parents, we selected for further analysis four F3 progeny that were chromosome 5 disomics (2R 2Y) and confirmed the expected diploid chromosome number by counting metaphase chromosomes (Table S1B).

### Genome Structural Integrity in Chromosome 5 Trisomics

Previous work with a trisomic tobacco line suggested that the chromosome present in triplicate was vulnerable to breakage [6]. Here we used array CGH to assess genome integrity in selected progeny of *Arabidopsis* triploids, including chromosome 5 trisomics from the F2 and F3 generations (Table S1). Array CGH can detect not only imbalances of intact chromosomes but also parts of chromosomes resulting from breakage, thereby revealing the approximate location of a breakpoint.

**Figure 3:**
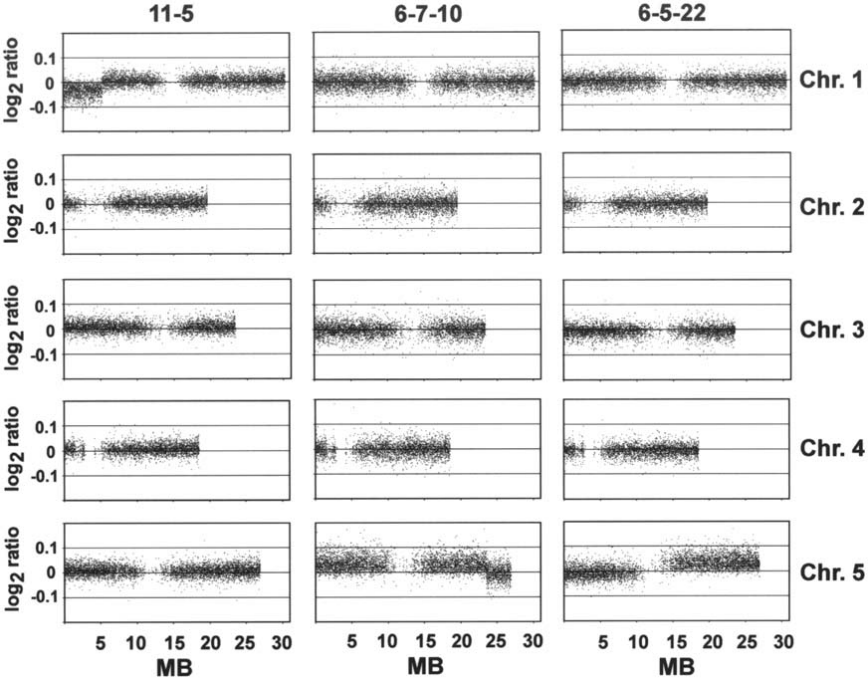
Chromosome breaks in trisomic and triploid plants. Array CGH detected truncated copies of chromosome 5 in two chromosome 5 trisomics (6-5-22 [potentially a secondary trisomic or isochromosome (2)] and 6-7-10), and a chromosome 1 truncation in a triploid plant (11-5). Each dot represents a probe set matching a unique gene model in the *Arabidopsis* genome. Identical chromosome copy numbers are indicated by a log2 ratio close to 0, while trisomy is characterized by the shift above the 0 baseline. Centromeres and pericentromeric heterochromatic regions are apparent by the areas deficient in dots.

The first chromosome break we detected was in a triploid plant from the F2 generation (11-5; Table S1), which contained a truncated copy of chromosome 1 lacking part of the top arm (Figures 2A and 3). The two trisomic F2 plants, 6-5 and 6-7, had structurally intact genomes as assessed by array CGH. In the F3 generation, however, we detected chromosome breaks in two trisomic plants (out of 26 tested by array CGH; Table S1B), one from each trisomic F2 parent. Both of these breaks affected the triplicated chromosome 5. In one case essentially the entire top arm of chromosome 5 was deleted (plant 6-5-22), suggesting a break around the centromere. In the second case, the break occurred in the vicinity of the *DsRed* transgene locus, such that the tip of the bottom arm of chromosome 5 was lost (plant 6-7-10) (Figure 2A and Figure 3).

Although derived from a relatively small sample size, these findings support the idea that trisomics show enhanced breakage of the chromosome present in triplicate and/or retention of a fractured chromosome when two intact copies are present. Because the truncated versions of chromosome 5 appeared in individual trisomic F3 progeny, they were likely generated during meiosis in the trisomic F2 parent. The possibility that breaks of the triplicated chromosome occur more frequently in somatic cells of trisomics than of diploids [23] can be studied in the future by performing single cell array CGH [24],[25].

Whether the trisomic plants containing truncated versions of chromosome 5 would transmit the broken chromosome to the next generation is not yet known. In a pilot study, a second generation chromosome 5 trisomic plant harbouring a break, again in the vicinity of the *DsRed* transgene locus (plant 12-16; Figure 2A), transmitted the truncated chromosome to trisomic progeny. However, array CGH of five trisomic progeny plants did not detect further deletions of chromosome 5 (data not shown). A more comprehensive study analyzing additional breakpoints in progeny plants across several generations might uncover evidence for progressive structural changes after formation of an initial break and reveal whether any specific DNA sequence features are associated with breakpoints. The current data suggest that repetitive regions, for example around the centromere and the *DsRed* transgene locus, which contains *lac* operator repeats [21],[22], are preferential sites of breakage in trisomics. The chromosome 1 break in the triploid plant 11-5 occurred in an intergenic, nonrepetitive region that does not contain conspicuous features.

### Transcript Expression Profiling

To assess the impact of chromosome 5 triplication on global gene expression, we carried out gene expression profiling using Affymetrix ATH1 microarrays, which report on about 21,000 *Arabidopsis* transcripts of the current TAIR genome annotation (v7). We were interested in comparing chromosome 5 trisomics and diploid plants with respect to the expression of genes on triplicated chromosome 5 (primary or cis effects) and the expression of genes on the four non-triplicated chromosomes (secondary or trans effects). All plants used for the transcriptome analysis (F2 trisomics 6-5, 6-7 and eight F3 progeny; F2 diploids 6-4, 7-2 and three F3 progeny) had intact genomes as assessed by array CGH (Table S1A,B).

**Figure 4:**
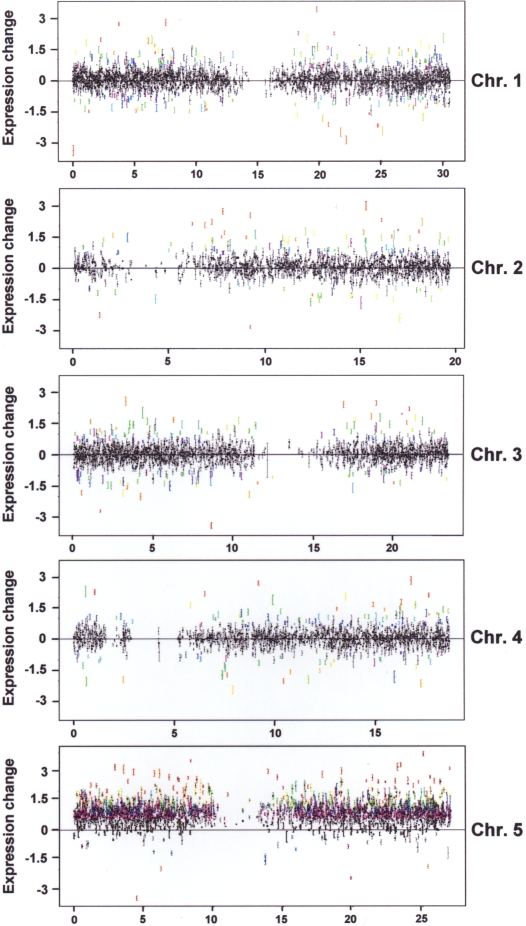
Distribution of significant expression changes across the five *Arabidopsis* chromosomes. Each transcript is represented by a mark and error bar. The *x*-axes correspond to the gene centre locations along the chromosomes, the *y*-axes show expression change, with positive values indicating increased expression in the trisomic plants. Rainbow colours report on relative significance (red/yellow is highest, blue/magenta is lowest). Genes on chromosome 5 that are dosage compensated are at the zero line; any gene significantly above is not dosage compensated. Lowly expressed genes are not included in these survey plots as their expression changes are more difficult to detect accurately (see Figure 5 and text for discussion).

Microarray hybridization signals not only showed a strong systemic effect for the trisomic chromosome 5 but also a wide range of clear trans effects for transcripts on the disomic chromosomes (Figure 4) consistent across the relatively large number of biological replicates analysed. It is noteworthy that many popular normalization transforms are not appropriate for data sets with large-scale expression level shifts as seen here because these violate underlying assumptions of many methods. The consequential distortions and signal dampening are illustrated for reference in the Supporting Information (Text S1) and Online Supplement (http://bioinf.boku.ac.at/pub/trisomy2008), where we also discuss alternative normalization methods ranging from popular established tools used in previous studies [17],[18] to specialized approaches such as exploiting CGH data as reference.

**Figure 5:**
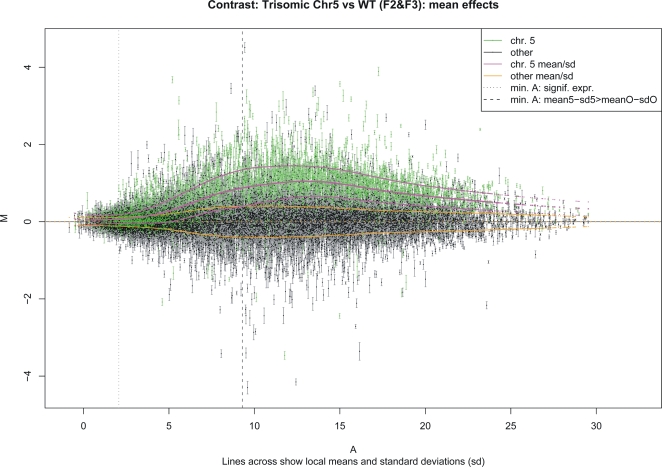
*M(A)* plot of the average expression differences *M* between chromosome 5 trisomic plants and disomics (*y*-axis) as a function of average expression *A* (*x*-axis). Transcripts on chromosome 5 are coloured green, and the intensity dependent trend plus/minus standard deviation is plotted in magenta. The trend for transcripts on other chromosomes is shown in orange. The centre trend orange dotted line traces the *x*-axis, reflecting that normalized expression differences for the other chromosomes average to zero. The dotted vertical line indicates the lowest expression intensity for which a statistically significant change could be detected with *p*<5% (Holm FWER). The dashed vertical line marks the intensity *A*_1+1_ where the lower magenta and the upper orange lines cross and the trends are separated by 1+1 standard deviations. The discussion of trends in the text focuses on transcripts to the right of the dashed line, where the survey will be most accurate (see Supplement for a discussion of this threshold). Normalized transformed values are shown, *i.e.*, scales are approximately logarithmic. As has been observed before for both trisomic samples and artificial spike-in data, the non-linear nature of the measurement system does not allow a direct interpretation of the expression difference measurements shown on the *y*-axis as calibrated log fold-change (*cf.* Figure 1 in [50]).

Observed expression levels of most transcripts on chromosome 5 reflected the dosage effect of its increased copy number in chromosome 5 trisomics, whereas most transcripts on other chromosomes did not change. Examination of expression differences as a function of average signal intensities in a traditional *M(A)*-plot, however, revealed an unexpected intensity dependence that has no biological explanation (Figure 5): Each transcript is represented by a dot and error bar, with the difference in expression (trisomics minus disomics) shown on the *y*-axis, and the average expression on the *x*-axis. Green marks the transcripts on chromosome 5. Magenta and orange trend lines respectively show the intensity dependence plus/minus one standard deviation for chromosome 5 and the other chromosomes. The deviation of the magenta centre trend line from a line parallel to the horizontal reflects the non-linear response of the detection system. The figure shows that differential expression is most accurately surveyed when using the microarray platform for sufficiently strongly expressed transcripts. We thus focused on the transcripts to the right of the dashed line (roughly half: 2,452/4,790 on chromosome 5 and 7,355/15,725 others), best reflecting the true trends for all the genes (*cf.* Text S1 and Online Supplement for discussion). Both average response and significant deviations from the chromosomal trends were studied.

Only a minor degree of dosage compensation was observed, with the percentage of genes on chromosome 5 classed as having similar expression levels in both trisomic and diploid plants ranging from 3% (by convex decreasing density estimate [26]) to 11–15% (89% differential expression for Benjamini-Yekutieli FDR *q*<5%). Interestingly, despite the increased gene dosage, 1% of transcripts on chromosome 5 had significantly lower expression levels than in the diploid. Whether the observed down-regulation is due to epigenetic silencing, altered transcription factor availability, or other mechanism is not yet known. The down-regulated genes, which are for the most part rather uniformly distributed along chromosome 5 (Figure 4), do not appear to have any conspicuous common features.

In contrast to the modest number of dosage-compensated and down-regulated genes, the highest proportion of chromosome 5 transcripts (86–88%) showed a significant increase in expression (partial or full dosage effect), reflecting the extra copy of chromosome 5 in the trisomics (88% significantly upregulated; 14% of expression changes below the trend; both with Benjamini-Yekutieli FDR *q*<5%). The expression increase of 12–13% of transcripts on chromosome 5 was even significantly above the average trend (hyper-dosage effect) for this chromosome (13% with Benjamini-Yekutieli FDR *q*<5%).

To verify this general trend also for chromosome 5 genes with lower expression levels, we used more sensitive quantitative RT-PCR (qRT-PCR) to quantify transcript levels of four moderately expressed genes on this chromosome, selected for their minimal variation during development (http://www.weigelworld.org/resources/microarray/AtGenExpress/) and five lowly expressed genes. Consistent with the general chromosome 5 trend, a higher steady-state transcript level in trisomics was indeed observed for the majority of these genes, confirming a dosage effect (Figures S1 and S2).

A different picture emerged for the secondary or trans effects on the other chromosomes: While the 12–13% ratio of transcripts up-regulated relative to the trend was similar, only 8–9% of transcripts on other chromosomes were significantly down-regulated, giving a strong 3∶2 skew favoring up-regulation *vs* down-regulation. Trans-effects were equally distributed across all chromosomes (Figure 4, Fisher's exact test, *p* = 33%), indicating that trisomy 5 has a genome-wide effect on gene expression.

Stress response genes and transcription factors were significantly overrepresented among the genes involved in trans-effects (Table 1). Indeed, the ten most-significant trans-effects included four transcription factors, of which three were strongly up-regulated (AGL19, ANAC019, AtMYB47) and one down-regulated (MEE3). The prominence of transcription factors in the strongest trans effects supports the gene balance hypothesis [2]. For the cis effects, genes involved in responses to abiotic or biotic stimulus and cell wall components were significantly affected whereas for dosage-compensated genes on chromosome 5, genes involved in structural roles and ribosome biogenesis were significantly over-represented (Table 1).

**Table 1:** GOslim categories significantly over-represented (odds ratio>1) or under-represented (odds ratio<1) in the test group relative to the entire chip (Fisher's exact test, Holm FWER<5%).

Trans-effects: genes differentially expressed		
Odds ratio	*p* value	Category
2.32	2.1×10^−7^	response to abiotic or biotic stimulus
2.23	0.000011	response to stress
2.18	0.000016	transcription factor activity
2.12	0.00003	other biological processes
0.63	0.0032	other intracellular components
0.60	0.0043	other cytoplasmic components
0.20	0.007	ribosome
3.72	0.0091	extracellular
2.75	0.014	cell wall
0.68	0.018	protein metabolism
1.56	0.022	transcription
0.33	0.037	nucleic acid binding
0.71	0.042	chloroplast

Cis-effects, genes differentially expressed relative to the chr. 5 trend		
Odds ratio	*p* value	Category
2.05	0.00087	response to abiotic or biotic stimulus
2.83	0.0056	cell wall
0.72	0.0083	unknown cellular components
0.00	0.0097	DNA or RNA metabolism
0.76	0.017	unknown biological processes
1.31	0.019	other membranes
3.43	0.024	other cellular components

Cis-effects, dosage compensated genes		
Odds ratio	*p* value	Category
5.07	1.0×10^−8^	structural molecule activity
4.77	5.9×10^−7^	ribosome
0.58	0.00028	unknown cellular components
3.03	0.00033	cytosol
0.10	0.0012	other molecular functions
1.66	0.0013	chloroplast
0.47	0.0028	protein binding
1.91	0.013	plastid

The first two test groups, for trans and for cis effects, consider genes differentially regulated relative to the average chromosomal trend. The third test group considers dosage compensated genes on the triplicated chromosome 5. Tests were conducted in the regime where the groups could accurately be delineated (strongly expressed genes, average expression A>A_1+1_, see Figure 5).

Changes in the expression of genes encoding transcription factors may alter the expression of numerous target genes and hence contribute to the genome-wide changes in expression observed in chromosome 5 trisomics. Similarly, changes in genes encoding epigenetic modifiers might also be expected to influence the expression of multiple target genes distributed throughout the genome. Chromosome 5 genes encoding known epigenetic modifiers showed the higher expression levels of the expected dosage effect in chromosome 5 trisomics. These include the DNA methyltransferases DRM2, DRM1, and MET1; the histone modifying enzymes HDA6 and SUVH4; and the SNF2-like chromatin remodeling protein DDM1 (Figure S3). In addition, epigenetic modifiers encoded on non-triplicated chromosomes were also involved in the trisomy 5 response. These include two genes on chromosome 2: *ROS1*, which encodes a DNA glycosylase-lyase protein involved in active demethylation of cytosines in DNA and hence acts antagonistically to MET1, DRM2 and DRM1 [27]; and *RDR5*, which encodes an RNA-dependent RNA polymerase related to those acting in RNAi-mediated pathways in plants [28] (Figure S4). Previous work has shown a link between components required for DNA methylation and those for active demethylation of DNA [29]. For example, in *met1* mutants, which have decreased levels of DNA methylation, *ROS1* expression is significantly reduced [29],[30]. One possibility is that the increased expression of DNA methyltransferases encoded on chromosome 5 might be counterbalanced by increased *ROS1* expression to maintain global DNA methylation at a level compatible with plant viability. Further work is needed to test this hypothesis.

In summary, transcript expression profiling by microarrays revealed that while the increased expression of the majority of transcripts (86–88%) on chromosome 5 reflected a partial, full, or hyper-dosage effect due to the triplication of this chromosome, there was a small set of transcripts (3–15%) for which there was evidence of dosage compensation. In contrast, there were 12–13% of transcripts across *all* chromosomes that were up-regulated with respect to their chromosomal neighborhoods. While there were at least as many transcripts (13–14%) on chromosome 5 down-regulated relative to the chromosome trend, down-regulation on other chromosomes was only observed for 8–9% of transcripts.

Generally elevated expression levels reflecting dosage effects for the triplicated chromosome, a genome-wide 3∶2 skew favoring up-regulation *vs* down-regulation in gene specific response, and dosage-compensation for some genes on chromosome 5 can together account for all these observations.

### Transcription of ROS1 and RDR5 in Other Trisomics

To determine whether the up-regulation of *ROS1* and *RDR5* in chromosome 5 trisomics is a generic response to an increased chromosome number or is specific for chromosome 5 trisomics, we used qRT-PCR to investigate expression of these genes in other F2 trisomics obtained from self-fertilization of the triploid F1 parents (Figure 2C; Table S1).

**Figure 6:**
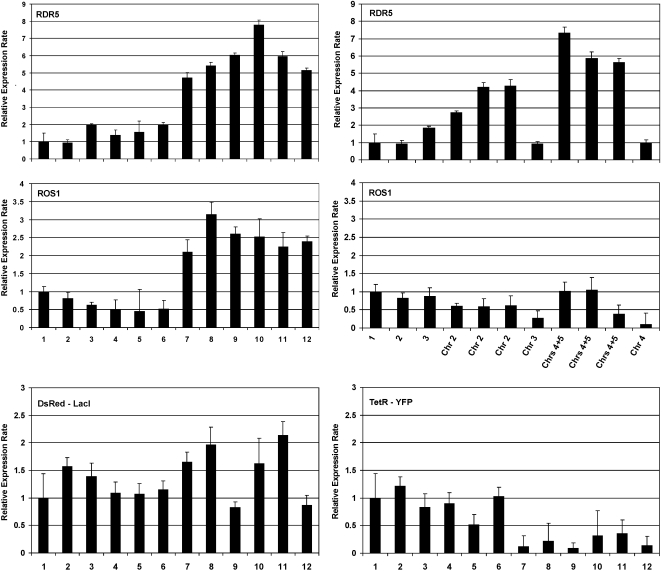
Quantitative RT-PCR. The relative expression levels of *RDR5* and *ROS1* were determined in six diploid plants (lanes 1-6; plants 6-4-2, 6-4-3, 7-2-1, 7-2-2, 7-2-3, 7-2-4) and six chromosome 5 trisomics (lanes 7–12; plants 6-5-6, 6-5-8, 6-7-19, 6-7-20, 6-7-21, 6-7-22) (left, top and middle) as well as in trisomics for other chromosomes (chr. 2, chr. 3, chr. 4) and double trisomics (chrs 4+5) (right, top and middle). The relative expression levels of the *DsRed-LacI* and *TetR-YFP* transgenes were compared in diploids (lanes 1–6) and chromosome 5 trisomics (lanes 7–12) (plant identities are the same as for *RDR5* and *ROS1*) (bottom left and right).

Despite their similar behaviour in individual chromosome 5 trisomics (Figure 6, top and middle, left, compare diploid lanes 1–6 with trisomic lanes 7–12) , *ROS1* and *RDR5* showed independent responses in other trisomics. For example, triplication of chromosome 2 (three plants available for testing) resulted in higher expression of *RDR5* at a level consistent with the increased gene dosage (Figure 6, top, right, lanes chr. 2) while *ROS1* expression was slightly below the diploid level, suggesting dosage compensation of this gene in the triplicated state (Figure 6, middle, right, lanes chr. 2). Both genes were sharply down-regulated in chromosome 3 and chromosome 4 trisomics, although only single plants were available for testing (Figure 6, top and middle, right, lanes chr. 3 and chr. 4). In three plants harbouring triplications of both chromosome 4 and chromosome 5 (double trisomics), an intermediate level of *ROS1* expression (around that observed in diploids) was observed (Figure 6, middle, right, lanes chrs. 4+5). By contrast, *RDR5* was expressed in the double trisomics at a level comparable to chromosome 5 single trisomics (Figure 6, top, compare lanes chrs. 4+5, right, with trisomic lanes 7–12, left). One interpretation of these results is that positive regulators of *ROS1* and *RDR5* are on chromosome 5, and in addition, a negative regulator of *ROS1* is on chromosome 4.

The data on *ROS1* and *RDR5* expression illustrate the complex variations in the expression of single genes in aneuploids of different chromosome constitutions. Genes encoding epigenetic modifiers can change expression independently, regardless of whether they are present on a numerically altered chromosome. These findings suggest that different aneuploidies might variably affect epigenetic mechanisms, creating diverse patterns of epigenetic modifications depending on the chromosome constitution. Additional work to determine genome-wide distributions of various epigenetic modifications in different aneuploids is required to test this conjecture.

### Expression of *DsRed-LacI* and *TetR-YFP* Transgenes on Chromosome 5

We also used qRT-PCR to examine the expression of *DsRed-LacI* and *TetR-YFP* transgenes, which are present on chromosome 5 but not represented on the ATH1 microarray. Interestingly, even though the *DsRed-LacI* and *TetR-YFP* transgenes are both transcribed by the cauliflower mosaic virus 35S promoter [21],[22], they respond differently to triplication of chromosome 5. The *TetR-YFP* gene was strongly down-regulated in chromosome 5 trisomics compared to diploids (Figure 6, bottom, right, diploid lanes 1–6, trisomic lanes 7–12). By contrast, the average expression of the *DsRed-LacI* gene remained at roughly the same level in both diploid and chromosome 5 trisomic plants, consistent with dosage compensation of this transgene when triplicated (Figure 6, bottom, left, diploid lanes 1–6, trisomic lanes 7–12). The expression of *Ds-Red-LacI* appears to display more plant-to-plant variability in trisomics than in diploids, however, suggesting a stochastic element to the dosage compensation mechanism (Figure 6, bottom, left, diploid lanes 1–6, trisomic lanes 7–12).

It is unknown why the two 35S promoter-driven transgenes reacted differently upon triplication of chromosome 5 nor is it clear why the *TetR-YFP* transgene undergoes such a steep reduction in expression when triplicated. Silencing and methylation of a transgene encoding neomycin phosphotransferase in tobacco was observed when the transgene locus was present on all three copies of a triplicated chromosome [6]. Both the *TetR-YFP* and *DsRed-LacI* transgene loci comprise complex inserts of the respective transgene construct [22]. The *TetR-YFP* transgene is integrated near a cluster of silent transposon-related sequences and tRNA genes (At5g20852 to At5g20858) that give rise to numerous small RNAs (http://mpss.udel.edu). By contrast, the *DsRed-LacI* transgene is inserted into two overlapping, moderately expressed protein-coding genes (At5g58140 and At5g58150) in a gene-rich region [21]. Perhaps the repetitive and silent genomic environment enhances silencing of the *TetR-YFP* transgene in trisomics. The basis of *TetR-YFP* silencing and whether repressive epigenetic modifications and/or small RNAs are involved remain to be determined. Although most down-regulated endogenous genes on triplicated chromosome 5 are not in repetitive regions, two of the most robustly down-regulated predicted genes (At5g35480, At5g35490; http://bioinf.boku.ac.at/pub/trisomy2008/nonorm2/down.cis.minA.ldiff.triVsWT.EBFWER.txt) are divergently transcribed from a common promoter and associated with transposon-related sequences and numerous small RNAs (http://mpss.udel.edu).

### 3D Arrangement of Fluorescent-Tagged Sites on Chromosome 5

The fluorescent-tagged sites on chromosome 5 are useful for identifying chromosome 5 trisomics at an early stage of development before the characteristic phenotype of trisomy 5 is visible. In addition, high resolution measurements of distances between *DsRed* and *YFP* transgene alleles can be made in interphase nuclei of living cells and subsequent 3D reconstructions of optical sections of nuclei can reveal the relative arrangements of the fluorescent tags. In a previous study of 16 different fluorescent-tagged sites distributed throughout the genome in diploid plants, random arrangements were observed in interphase nuclei of root cells. There was no indication of allelic pairing (defined as an inter-allelic distance of ≤ 0.5 µm) or for preferential associations of ectopic chromosome sites in diploid plants [21]. In the present study, we compared chromosome 5 trisomics with triploids, both of which have three YFP dots and three DsRed dots in the context of a chromosomally unbalanced or balanced genome, respectively (Figure 1). We examined whether the extra copy of chromosome 5 in trisomics produced any distinctive arrangements of chromosome 5 fluorescent tags that differed from those observed in the triploid genome.

Six distances – connecting the three YFP dots and the three DsRed dots – were measured in selected root nuclei in which fluorescent signals were visible (Figure S5). In sibling triploid and trisomic seedlings of the F2 generation, the distances between the YFP dots and DsRed dots usually differed within a given nucleus and considerable inter-nuclear variability in distance measurements was observed for both fluorescent tags (Table S2A,B). Thus, in both trisomics and triploids, chromosome 5 fluorescent tags display similar random arrangements. In trisomics, however, we observed an increased incidence of inter-allelic distances around 0.5 µm (Table S2B). Although these results might suggest enhanced allelic pairing in trisomics, they could also reflect the generally smaller inter-allelic distances in these plants (Table S2), which in turn is probably due to smaller nuclei in trisomics than in triploids [21]. The possibility of enhanced allelic associations in trisomics was supported, however, by 3D reconstructions of nuclei, which indicated that two of the three alleles of either *DsRed* or *YFP* were more likely to be close to each other in trisomics than in triploids (group I, Table S2; Figure S6). A similar trend was observed in trisomic F3 progeny; however, analysis of these plants was compromised by problems with epigenetic silencing of the *LacI-DsRed* and *TetR-YFP* transgenes and by the lack of F3 triploid siblings for comparison (Table S1B and data not shown).

Although the analysis has involved a limited number of root cell nuclei, it appears that the presence of an extra chromosome 5 in unbalanced trisomics does not substantially alter the interphase arrangement of chromosome 5 fluorescent tags as compared to those observed in chromosomally balanced triploids. A subtle difference, however, may be a slightly enhanced tendency for two copies of the triplicated chromosome to be more closely apposed, at least partially along their lengths, in trisomics than in triploids. This possibility can be studied in the future with a larger set of trisomic plants and the use of emerging strategies that minimize silencing of the reporter transgenes [22].

### General Summary and Conclusions

Our studies on the influence of chromosome 5 triplication on chromosome structural stability, gene expression, and interphase arrangement of chromosome 5 fluorescence tags in *Arabidopsis* have demonstrated that trisomy 5 disrupts the genome in a number of ways:

1. Chromosome structural stability: Truncated derivatives of the triplicated chromosome 5 were regularly observed in trisomic plants. The triplicated chromosome may be vulnerable to breakage, particularly in vicinity of repetitive regions, and a truncated chromosome is more likely to be retained when two intact copies are present. The possibility of structural as well as numerical deviations in aneuploids underscores the need to perform array CGH for proper analysis and intepretation of the transcriptome data [31]. The formation and inheritance of chromosome structural variants in aneuploids might have evolutionary implications if restructured chromosomes are transmitted to progeny and eventually fixed in the population [32]. Enhanced structural instability of aneuploid genomes in somatic cells could have relevance for human cancer cells, which display progressive chromosome numerical and structural changes as the tumour evolves [7],[23].

2. Complex changes in gene expression: The transcriptome analysis revealed that the expression of many genes is affected in chromosome 5 trisomics, primarily on the triplicated chromosome (cis effects) but also on non-triplicated chromosomes (trans effects). Most genes on chromosome 5 genes showed higher expression reflecting a dosage effect, but cases of apparent dosage compensation and even down-regulation were also observed. Genes involved in responses to stress and other stimuli were over-represented among genes differentially regulated relative to the average chromosome trends, and transcription factors were over-represented in the trans effects. The use of qRT-PCR to analyze expression of single genes demonstrated variable expression depending on the chromosome number and constitution, and on the features of individual genes: As shown with the epigenetic regulators *ROS1* and *RDR5*, genes on the same chromosome can vary independently in their expression in different trisomics. In addition, genes under the control of the same promoter can vary in their response to triplication, as indicated by the two 35S promoter-driven transgenes, *TetR-YFP* and *DsRed-LacI*, on chromosome 5. The observed variations in gene expression probably depend on multiple factors including, but not limited to, changes in the dosages of regulatory molecules and epigenetic factors, and sensitivity of repetitive regions to copy number changes and gene silencing mechanisms. Transcriptional changes resulting from aneuploidy must be described in terms of chromosomes and/or chromosome regions that are numerically altered and whether changes in expression are in cis or trans regions. Clearly, the choice of microarray data analysis methods has a substantial impact on results and, in particular, normalization methods that are robust to large-scale shifts in gene expression need to be applied in studies of aneuploidy. Although not studied here, cell and tissue-type differences in gene expression in a given aneuploid might also be expected [15].

3. 3D organization of fluorescent-tagged sites: Overall, chromosomally unbalanced trisomics and balanced triploids display equally random interphase arrangements of fluorescent tagged sites on chromosome 5; however, there may be a slight tendency for two transgene alleles on the triplicated chromosome to be more closely associated in trisomics than in triploids. If such associations occur regularly in trisomics, they might help to induce dosage compensation mechanisms [33] or spatially compensate for the extra chromosome in interphase nuclei.

Aneuploidy is usually studied for its developmentally detrimental or pathological consequences but it also may be important in normal contexts. Recent work has identified a significant fraction of aneuploid cells in the normal brain although their physiological significance is not yet known [34]. Given the strong effect of aneuploidy on global gene expression patterns, it is conceivable that the formation of aneuploid neurons increases the phenotypic variability of these cells and their capacity to perform diverse neural functions.

## Materials and Methods

### Plant Material

The plant material in all experiments was *Arabidopsis thaliana* landrace Col-0 (the accession used for the design of the ATH1 array). The transgenic line with YFP and DsRed fluorescent tags on chromosome 5 was described previously [21]. Seeds were germinated on sterile, solid Murashige and Skoog medium in plastic petri dishes. Root nuclei in living seedlings were monitored for YFP and DsRed fluorescence signals as detailed in previous reports [21],[22]. Seedlings were then transferred to pots containing a mixture of Huminsubstrat N3 and Vermiculit Nr.2 (2∶1 v/v) (purchased from a local supplier), and placed in a culture room with natural light (3000 lux). The photoperiod was 16 h and temperature was maintained at 23°C. Single leaves were cut from the plants at a stage of approximately ten rosette leaves (>1 cm in length), except for plants with extreme aberrant phenotypes, which late were found to contain an extra copy of chromosome 1. The first cut leaf was selected for RNA and the second for DNA isolation in order to minimize wounding effects.

### Production of Tetraploids, Metaphase, and Interphase Chromosome Analysis

Seedlings were treated with colchicine to produce tetraploid progeny according to an unpublished protocol (Ramon Angel Torres Ruiz, personal communication). Metaphase chromosome counts were performed using pistil material as described in protocols 5.2 and 5.3 in a previous publication [35].

Inter-allelic distances and 3D arrangements of fluorescent tagged sites on chromosome 5 in root interphase nuclei of living, untreated seedlings were determined using fluorescence microscopy as described previously [21],[22]. The tagged sites harbor transgene complexes that encode repressor protein-fluorescent protein fusions proteins (either Tet-YFP or DsRed-LacI) as well as arrays of either *tet* or *lac* operator repeats, to which the respective repressor protein-fluorescent protein fusion protein can bind [21],[22].

### Comparative Genome Hybridization with Microarrays

Isolation of genomic DNA (DNeasy mini kit, Qiagen, Hilden, Germany), biotin labelling of DNA (BioPrime DNA labelling, Invitrogen, Lofer, Austria), and gDNA hybridization were performed as described [36]. The DNA concentration was quantified by spectrophotometry (Nanodrop ND-1000; Peqlab, Erlangen, Germany) and adjusted for gDNA hyridization to 15 µg. ATH1 microarrays were scanned with an Affymetrix GC3000 system and analysed with GCOS version 1.4 (Affymetrix, High Wycombe, U.K.). For chromosome copy number variation the disomic transgenic plant, from which all triploid, tetraploid, and trisomic plants were derived, served as the reference microarray. The array signals from the derived plants were scaled in GCOS and compared to the diploid progenitor. Extra chromosomes or chromosomal deletions were then identified after sorting for probe sets with a “change p-value” call “Increase” for supernumerical chromosomes or a “Decrease” call for deletions. In all cases the default settings were chosen. After excluding probe sets matching to several gene models (TAIR7) the remaining probe sets were mapped to the *Arabidopsis* chromosomes (chromosome map tool at www.arabidopsis.org). Typically, extra chromosomes are identified by mapping 95% to 98% of probe sets with an “Increase” call to a unique chromosome e.g. chromosome 5 in case of chromosome 5 trisomy.

### Mapping Deletion to Chromosomes

Microarrays were normalized and log transformed by the RMAExpress0.5 tool (http://rmaexpress.bmbolstad.com/). The log ratios of the signal values were mapped to their chromosomal position. Data on probe set location was also extracted from TAIR v7 (see microarray data analysis section). Only probe sets matching to a unique gene model (TAIR7) were selected.

### Quantitative Real-Time PCR Analysis

RNA extraction (RNeasy mini kit, Qiagen, Hilden, Germany) and cDNA synthesis (RevertAid H Minus First strand cDNA synthesis kit, MBI Fermentas, St. Leon-Rot, Germany) were performed as described previously [37]. The cDNA was diluted to 75 µl with DEPC-treated double distilled water, and 2 µl was used in a 20 ul PCR reaction. The mixture was set up with 10 µl of QuantiFast SYBR Green PCR (Qiagen, Hilden, Germany), 2 µl cDNA, and 2 µl of each primer (1 µM final concentration). PCR was performed after a preincubation as suggested by the supplier (95° C for 5 min) by 40 two-step cycles of denaturation at 95° C for 10 s, and annealing/extension at 60° C for 30 s. The comparative threshold cycle (Ct) method was used to determine relative RNA levels (User Bulletin no. 2, Applied Biosystems). GAPC-2 (At1g13440) was chosen as the internal reference gene (see also [38] for a comprehensive analysis of reference genes), and expression levels are relative to a randomly chosen disomic plant. Sequence of the primer sets are shown in Table S3.

### Transcriptome Analysis

Total RNA was extracted from rosette leaves (>1 cm in length) using an RNeasy mini kit (Qiagen, Hilden, Germany). Transcriptomes were analysed using 1 µg of total RNA as starting material. Targets were prepared with the one-cycle cDNA synthesis kit followed by biotin-labelling with the IVT labelling kit (GeneChip One-cycle target labelling and control reagents, Affymetrix, High Wycombe, U.K.) and hybridized for 16 h as recommended by the supplier (Gene expression analysis manual, Affymetrix). All transcriptome data (CEL and CHP files) were submitted to a public repository database (http://www.ebi.ac.uk/microarray/, ArrayExpress accession number: E-MEXP-1454.

### Microarray Data Analysis

#### Low-Level Analysis and Transforms

A total of 19 samples from 15 individual plants (2×2 trisomics|F2, 2×2 disomics|F2, 8 trisomics|F3, 3 disomics|F3 was hybridized to Affymetrix ATH1-type GeneChips and scanned as described above. Low-level CEL-file analysis included re-assignment of probes to a current TAIR genome annotation, removal of probe-sequence specific effects, chip-to-chip normalization, and a robust expression signal summary of probe sets using a multi-chip model to down-weight random outlier probes.

The original ATH1 design comprised probe sets for 22,810 transcripts. Probe set size ranged from 8 to 20 probes per target, with a mean of 11.0±0.3. Depending on the target organism, however, the ongoing improvements in genome annotation can considerably affect differential expression estimates for 30–40% of all the targets of an Affymetrix chip [39]. The necessary re-assignment and re-annotation of probes consistent with a current genome annotation (TAIR v7) resulted in 21,089 probe sets (custom assignment v10). Data on transcript chromosomal locations and start and end coordinates were also extracted from TAIR for probe-set annotation. Further examination revealed several probe sets with probes perfectly matching multiple chromosomal locations, which we wanted to exclude for this study. This finally left 20,515 probe sets ranging in size from 3 to 32 probes per target, with a mean of 10.8±1.4.

Probe specific effects have been fit using an Empirical Bayes ‘affinities’ model for removing both probe-specific background and adjusting perfect-match signal intensities for probe-specific affinities [40]. Probe level signals were conservatively normalized for different backgrounds and overall hybridization intensities of individual chips using an iterative 20%-trimmed least squares fit of a generative model with additive-multiplicative noise [41]. This approach is robust both to outliers and to systemic large-scale shifts, as could be seen from estimating transform parameters from all data or only from genes not on chromosome 5 (data not shown). The variance-stabilizing generalized log transform for this model was calibrated for asymptotic equivalence to a standard log_2_ transformation. We refrained from further transforms in a first examination of data characteristics. As can be seen from Figure 5 the conditions for many popular more aggressive normalization methods (such as quantile–quantile normalization or *M(A)*-Loess) were not satisfied.

Transcript expression estimates were obtained by robust fits of linear multi-chip probe level intensity models [42].

A number of diagnostic plots are provided in the Online Supplement (e.g. pair-wise *Q–Q* and *M(A)*, spatial residual trends). We also investigated the effect of alternative normalization options, including standard methods like quantile normalization and specialized approaches like attempting to exploit CGH hybridization signals for normalization. Results corroborate our choice of conservative normalization. See Methods section of Text S1 and the Online Supplement at http://bioinf.boku.ac.at/pub/trisomy2008/.

#### Analysis of Differential Expression

For every gene, linear models were fit to obtain a contrast between chromosome 5 trisomic and normal diploids, correctly weighted for unbalanced design and independently for F2 and F3 progeny. We then studied the average contrast for F2 and F3 progeny.

In an examination of chromosome-wide trends, instead of the constant increase in expression expected for transcripts on chromosome 5, a clear and strong intensity dependence could be observed, which cannot be explained by biological effects. Figure 5 shows expression change as a function of average expression in a standard *M(A)*-plot. Transcripts on chromosome 5 are coloured green, and the intensity dependent trend plus/minus standard deviation is plotted in magenta. The trend for other transcripts is shown in orange. Intensity-local trend lines and standard deviations were computed in R by a Loess smoother with span 0.4. The increased expression of transcripts on chromosome 5 becomes clearer with higher average expression (*x*-axis), with the trends being separated by 1+1 standard deviations where the lower magenta and the upper orange lines cross. We denote this intensity by *A*_1+1_, marked by a vertical dashed line. The separation continues to grow with the average intensity, peaks, and then decreases but without falling below the amount at *A*_1+1_. As a consequence, an analysis of expression changes will be most accurate for *A*>*A*_1+1_. For our analysis of trends we therefore focused on this regime.

For an analysis of deviations from the average trend of transcripts on chromosome 5, we performed a calibration by subtracting the average trend as fitted by the Loess smoother. Deviations could then be tested as deviations from zero (see Results section of the Online Supplement).

We tested for differential expression of each gene applying an Empirical Bayes regularized *t*-test [43]. Unless mentioned otherwise in the text, *p*-values used in the generation of lists and graphs were corrected for multiple testing using the conservative approach by Holm [44], providing strong control of the family wise error rate (FWER), when assessing change, and by the more powerful approach of Benjamini and Yekutieli [45], providing strong control of the false discovery rate (FDR), in the case of testing for non-change, each with a threshold of 5%, yielding conservative conclusions in either case. Trend estimates used the Benjamini-Yekutieli (BY) approach, considering the 5% upper bound of the FDR to calculate a lower bound of the detected true positive range.

For an overview of functional gene categories affected current ‘GOslim’ annotation was extracted from TAIR, v.2007-12-29 [46], and subset enrichment tested for significance (Fisher's exact test, Holm FWER *p*<5%). Contingency tables are available from the Results section of the Online Supplement at http://bioinf.boku.ac.at/pub/trisomy2008/.

## Supporting Information

Figure S1 qRT-PCR of low to moderately expressed genes on chromosome 5.
(0.06 MB DOC)

Figure S2 qRT-PCR of low expressed genes on chromosome 5.
(0.08 MB DOC)

Figure S3 Chromosome 5 calibrated cis effects.
(0.23 MB DOC)

Figure S4 Trans effects on expression of genes on chromosome 2.
(0.20 MB DOC)

Figure S5 Examples of connected YFP and DsRed dots for measurements of interallelic distances in three dimensions.
(0.04 MB PDF)

Figure S6 Boxplot of normalized shortest interallelic distance.
(0.03 MB DOC)

Table S1 List of plants.
(0.18 MB DOC)

Table S2 Interallelic distance measurements.
(0.36 MB DOC)

Table S3 Primers.
(0.03 MB DOC)

Text S1 Supporting information text.
(0.27 MB PDF)

## References

[1] Birchler JA, Yao H, Chudalayandi S (2007). Biological consequences of dosage dependent gene regulatory
systems.. Biochim Biophys Acta.

[2] Birchler JA, Veitia RA (2007). The gene balance hypothesis: from classical genetics to modern
genomics.. Plant Cell.

[3] Duesberg P (2007). Chromosomal chaos and cancer.. Sci Am.

[4] Jones PA, Baylin SB (2002). The fundamental role of epigenetic events in cancer.. Nat Rev Genet.

[5] Esteller M (2007). Cancer epigenomics: DNA methylomes and histone-modification maps.. Nat Rev Genet.

[6] Papp I, Iglesias VA, Moscone EA, Michalowski S, Spiker S (1996). Structural instability of a transgene locus in tobacco is
associated with aneuploidy.. Plant J.

[7] Matzke M, Mette MF, Kanno T, Matzke AJM (2003). Does the intrinsic instability of aneuploid genomes have a causal
role in cancer?. Trends Genet.

[8] Schneider R, Grosschedl R (2007). Dynamics and interplay of nuclear architecture, genome
organization, and gene expression.. Genes Dev.

[9] Hattori M, Fujiyama A, Taylor TD, Watanabe H, Yada T (2000). The chromosome 21 mapping and sequencing consortium.. Nature.

[10] Semple C (2004). Deep genomics in shallow times: the finished sequence of human
chromosomes 13 and 19.. Eur J Hum Genet.

[11] Hernandez D, Fisher EMC (1999). Mouse autosomal trisomy; two's company, three's
a crowd.. Trends Genet.

[12] Ait Yahya-Graison E, Aubert J, Dauphinot L, Rivals I, Prieur M (2007). Classification of human chromosome 21 gene expression variations
in Down syndrome: impact on disease phenotypes.. Am J Hum Genet.

[13] FitzPatrick DR (2005). Transcriptional consequences of autosomal trisomy: primary gene
dosage with complex downstream effects.. Trends Genet.

[14] Mao R, Wang X, Spitznagel EL, Frelin LP, Ting JC (2005). Primary and secondary transcriptional effects in the developing
Down syndrome brain and heart.. Genome Biology.

[15] Li CM, Guo M, Salas M, Schupf N, Silverman W (2006). Cell type-specific over-expression of chromosome 21 genes in
fibroblasts and fetal hearts with trisomy 21.. BMC Medical Genetics.

[16] Guo M, Birchler JA (1994). Trans-acting dosage effects on the expression of model gene
systems in maize aneuploids.. Science.

[17] Makarevitch I, Phillips RL, Springer NM (2008). Profiling expression changes caused by a segmental aneuploid in
maize.. BMC Genomics.

[18] Torres EM, Sokolsky T, Tucker CM, Chan LY, Boselli M, Dunham MJ, Amon A (2007). Effects of aneuploidy on cellular physiology and cell division in
haploid yeast.. Science.

[19] Matzke M, Mittelsten Scheid O, Allis CD, Jenuwein T, Reinberg D (2007). Epigenetic regulation in plants.. Epigenetics..

[20] Rédei GP, Koncz C, Koncz C, Chua N.-H-, Schell J (1992). Classical mutagenesis.. Methods in Arabidopsis Research..

[21] Matzke AJM, Huettel B, van der Winden J, Matzke M (2005). Use of two-color fluorescent-tagged transgenes to study
interphase chromosomes in living plants.. Plant Physiol.

[22] Matzke AJM, Huettel B, van der Winden J, Matzke MA (2008). Fluorescent transgenes to study interphase chromosomes in living
plants.. Methods Mol Biol.

[23] Nowell PC (1976). The clonal evolution of tumor cell populations.. Science.

[24] Le Caignec C, Spits C, Sermon K, De Rycke M, Thienpont B (2006). Single-cell chromosomal imbalances detection by array CGH.. Nucl Acids Res.

[25] Geigl JB, Speicher MR (2007). Single-cell isolation from cell suspensions and whole genome
amplification from single cells to provide templates for CGH analysis.. Nat Protocols.

[26] Ferkingstad E, Langaas M, Lindqvist B (2005). Estimating the proportion of true null hypotheses, with
application to DNA microarray data.. J Royal Statistical Society B.

[27] Zhu J, Kapoor A, Sridhar VV, Agius F, Zhu J-K (2007). The DNA glycosylase/lyase ROS1 functions in pruning DNA
methylation patterns in *Arabidopsis*.. Curr Biol.

[28] Wassenegger M, Krczal G (2006). Nomenclature and functions of RNA-directed RNA polymerases.. Trends Plant Sci.

[29] Huettel B, Kanno T, Daxinger L, Aufsatz W, Matzke AJ, Matzke M (2006). Endogenous targets of RNA-directed DNA methylation in Pol IV in
*Arabidopsis*.. EMBO J.

[30] Mathieu O, Reinders J, Caikovski M, Smathajitt C, Paszkowski J (2007). Transgenerational stability of the *Arabidopsis*
epigenome is coordinated by CG methylation.. Cell.

[31] Zanazzi C, Hersmus R, Veltman IM, Gillis AJM, van Drunen E (2007). Gene expression profiling and gene copy-number changes in
malignant mesothelioma cell lines.. Genes Chrom Cancer.

[32] Matzke M, Mittelsten Scheid O, Matzke AJM (1999). Rapid structural and epigenetic changes in polyploidy and
aneuploid genomes.. BioEssays.

[33] De Laat W, Grosveld F (2007). Inter-chromosomal gene regulation in the mammalian cell nucleus.. Curr Opin Genet Devel.

[34] Kingsbury MA, Yung YC, Peterson SE, Westra JW, Chun J (2006). Aneuploidy in the normal and diseased brain.. Cell Mol Life Sci.

[35] Schwarzacher T, Heslop-Harrison P (2000). Practical *in situ* hybridization.

[36] Borevitz J (2006). Genotyping and mapping with high-density oligonucleotide arrays.. Methods Mol Biol.

[37] Kanno T, Huettel B, Mette MF, Aufsatz W, Jaligot E (2005). Atypical RNA polymerase subunits required for RNA-directed DNA
methylation.. Nat Genet.

[38] Czechowski T, Stitt M, Altmann T, Udvardi MK, Scheible WR (2005). Genome-wide identification and testing of superior reference
genes for transcript normalization in *Arabidopsis*.. Plant Physiol.

[39] Dai M, Wang P, Boyd AD, Kostov G, Athey B (2005). Evolving gene/transcript definitions significantly alter the
interpretation of GeneChip data.. Nucl Acids Res.

[40] Wu Z, Irizarry RA, Gentleman R, Martinez Murillo F, Spencer F (2004). A Model Based Background Adjustment for Oligonucleotide
Expression Arrays.. J. Am. Stat. Assoc.

[41] Huber W, von Heydebreck A, Sültmann H, Poustka A, Vingron M (2002). Variance stabilization applied to microarray data calibration and
to the quantification of differential expression.. Bioinformatics.

[42] Bolstad B (2004). Low Level Analysis of High-density Oligonucleotide Array Data:
Background, Normalization and Summarization.. http://bmbolstad.com/.

[43] Smyth G K (2004). Linear models and empirical Bayes methods for assessing
differential expression in microarray experiments..

[44] Holm S (1979). A simple sequentially rejective multiple test procedure.. Scand J Statist.

[45] Benjamini Y, Yekutieli D (2001). The control of the false discovery rate in multiple testing under
dependency.. Ann Stat.

[46] Berardini TZ, Mundodi S, Reiser R, Huala E, Garcia-Hernandez M (2004). Functional annotation of the *Arabidopsis* genome
using controlled vocabularies.. Plant Physiol.

[47] Henry IM, Dilkes BP, Young K, Watson B, Wu H, Comai L (2005). Aneuploidy and genetic variation in the *Arabidopsis
thaliana* triploid response.. Genetics.

[48] Khush GS (1973). Cytogenetics of Aneuploids.

[49] Henry IM, Dilkes BP, Comai L (2007). Genetic basis for dosage sensitivity in *Arabidopsis
thaliana*.. PLoS Genetics.

[50] Irizarry RA, Cope LM, Wu Z (2006). Feature-level exploration of a published Affymetrix GeneChip
control dataset.. Genome Biology.

