# Supplementary material for: Effects of Aneuploidy on Genome Structure, Expression, and Interphase Organization in Arabidopsis thaliana
Source: PLoS Genet. 2008 Oct 17;4(10):e1000226. doi: 10.1371/journal.pgen.1000226 (PMC2562519; doi:10.1371/journal.pgen.1000226)
Supplement: Figure S1 — qRT-PCR of low to moderately expressed genes on chromosome 5. (0.06 MB DOC) [file pgen.1000226.s001.doc]

**
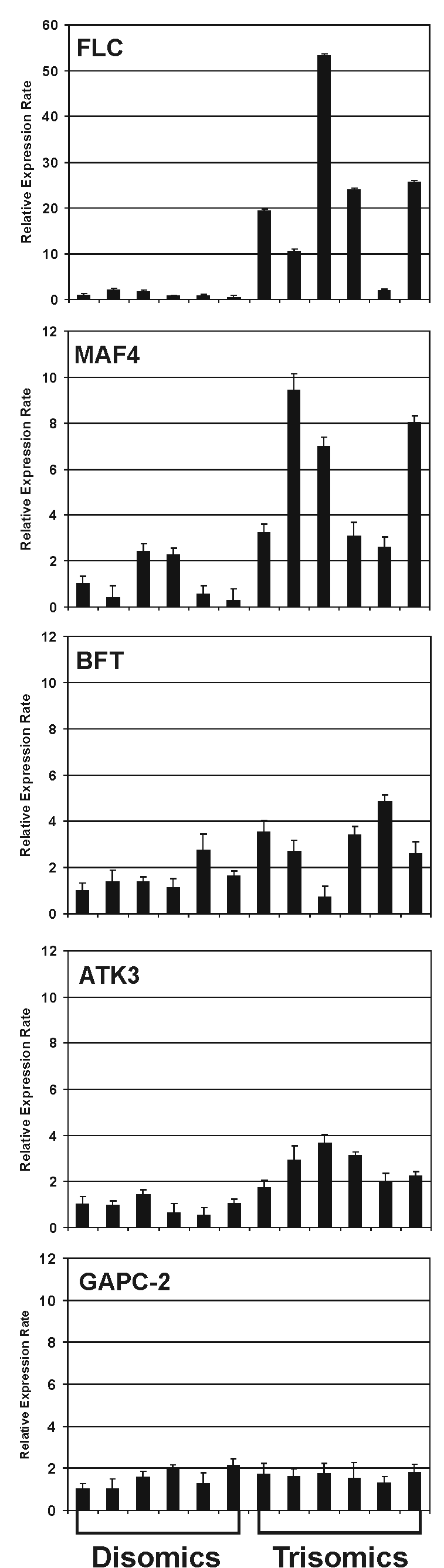
**

**Fig. S1. Quantitative RT-PCR of low to moderately expressed genes on Chr 5.**

Genes with an average expression below a threshold where chr. 5 genes were easily distinguished from other genes (genes to the left of the dashed line in Fig. 5) were selected that on the array showed upregulation relative to the chromosome trend (FLC and MAF4: log_2_ average expression = 5.5; BFT and ATK3: log_2_ average expression ≅ 2.2, Fig. 5). For all cases an increased gene expression was observed at a level reflecting a hyper dosage effect. The relative expression levels were determined in six diploid (disomics; lanes 1-6) and six chromosome 5 trisomic plants (trisomics; lanes 7–12). Expression was normalized to a randomly selected disomic plant and is shown on a linear scale. In contrast to chr. 5 genes, GAPC-2 (chr. 1) expression was stable in plants investigated irrespective of their chromosome number. UPL7 (At3g53090) was chosen as the internal reference gene (log_2_ average expression = 12.5, Fig. 5).

FLC (FLOWERING LOCUS C), MAF4 (MADS-box containing FLC paralog), BFT (brother of FT and TFL1 protein), ATK3 (ARABIDOPSIS THALIANA KINESIN 3).
