# Supplementary material for: Effects of Aneuploidy on Genome Structure, Expression, and Interphase Organization in Arabidopsis thaliana
Source: PLoS Genet. 2008 Oct 17;4(10):e1000226. doi: 10.1371/journal.pgen.1000226 (PMC2562519; doi:10.1371/journal.pgen.1000226)
Supplement: Figure S2 — qRT-PCR of low expressed genes on chromosome 5. (0.08 MB DOC) [file pgen.1000226.s002.doc]

**
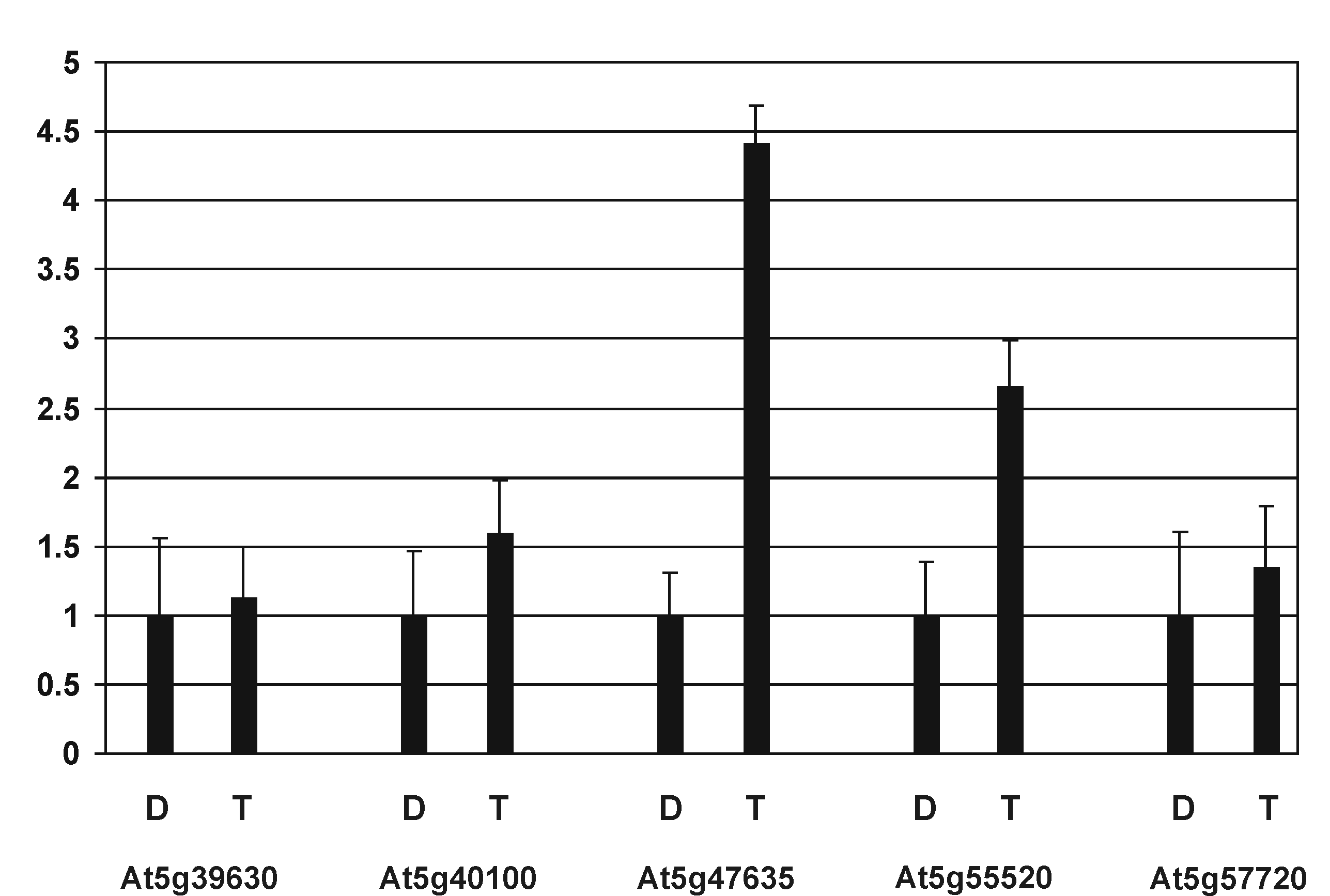
**

**Fig. S2. Quantitative RT-PCR of low expressed genes on Chr.5.**

The relative expression levels of genes with no significant expression differences from the chromosome trend deduced by microarray analysis and average expression below the dotted line "min. A" in Figure 5. The average effect for five plants per gene is shown on a linear scale: diploid plants (D), chromosome 5 trisomics (T). In chromosome 5 trisomic plants higher expression was observed in all cases and gene expression shows a partial, full, or hyper dosage effect (the expected full dosage effect of an 1.5-fold increase in trisomics is seen for At5g40100 and At5g57720). UPL7 (At3g53090) was chosen as the internal reference gene. Error bars: SD +/- mean.
