# Supplementary material for: Effects of Aneuploidy on Genome Structure, Expression, and Interphase Organization in Arabidopsis thaliana
Source: PLoS Genet. 2008 Oct 17;4(10):e1000226. doi: 10.1371/journal.pgen.1000226 (PMC2562519; doi:10.1371/journal.pgen.1000226)
Supplement: Figure S3 — Chromosome 5 calibrated cis effects. (0.23 MB DOC) [file pgen.1000226.s003.doc]

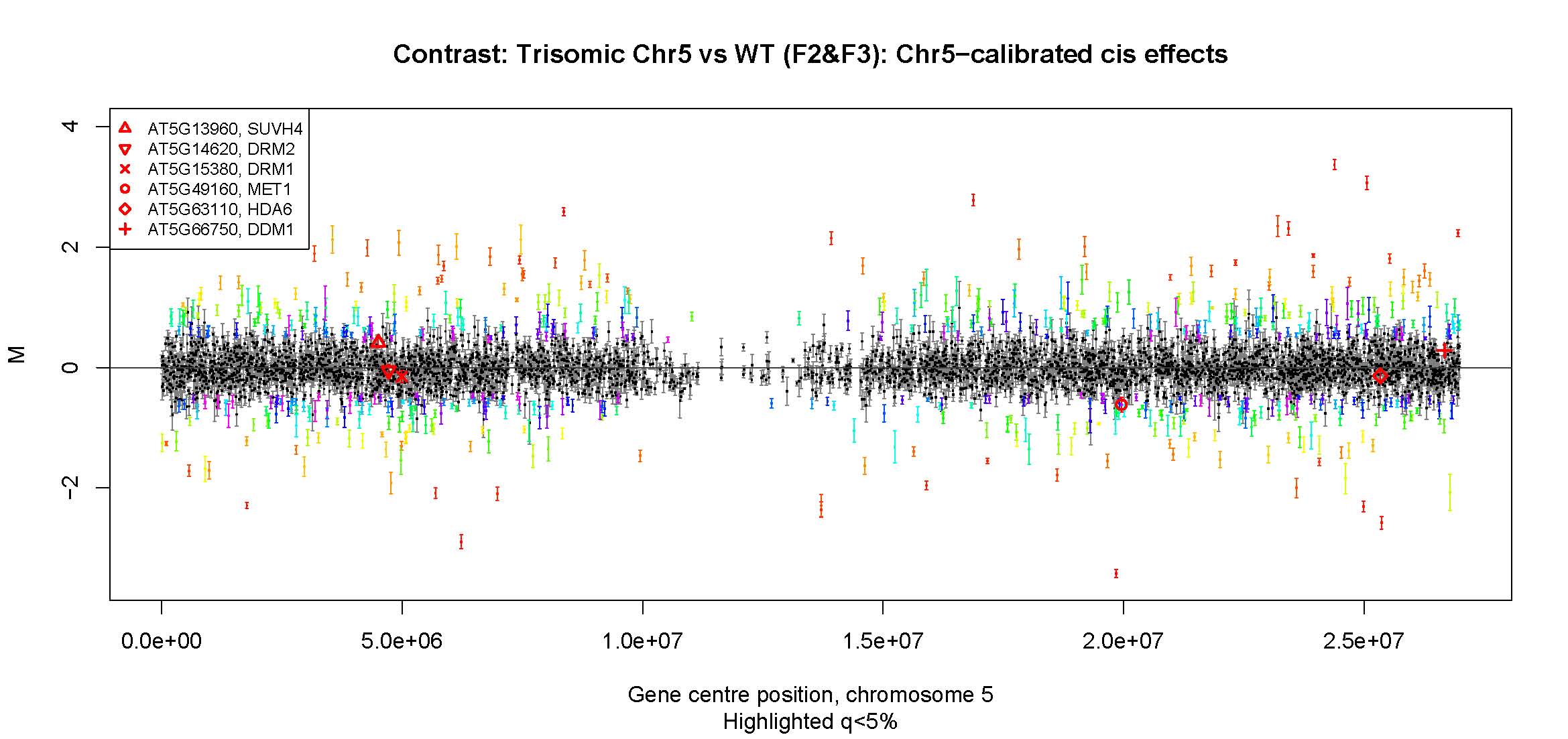


**Figure S3. Huettel et al.**

Chromosome 5 calibrated cis effects in *Arabidopsis* plants trisomic for chromosome 5.

Differential gene expression relative to the cis trend is plotted across the chromosome and each transcript is shown with a mark and an error bar. The *x*-axis corresponds to gene locations along the chromosome. The *y*-axis shows expression change, with positive values reporting expression higher than expected in the trisomic plants. Rainbow colours indicate relative significance (red/yellow is highest, blue/magenta is lowest). Most genes on chromosome 5 show the expected increased expression level. Only a minority of genes is below the general trend and therefore dosage compensated or down-regulated. Genes encoding six epigenetic modifiers located on the triplicated chromosome 5 are highlighted by red symbols. All six follow the general trend of increased expression.
