# Supplementary material for: Effects of Aneuploidy on Genome Structure, Expression, and Interphase Organization in Arabidopsis thaliana
Source: PLoS Genet. 2008 Oct 17;4(10):e1000226. doi: 10.1371/journal.pgen.1000226 (PMC2562519; doi:10.1371/journal.pgen.1000226)
Supplement: Figure S4 — Trans effects on expression of genes on chromosome 2. (0.20 MB DOC) [file pgen.1000226.s004.doc]

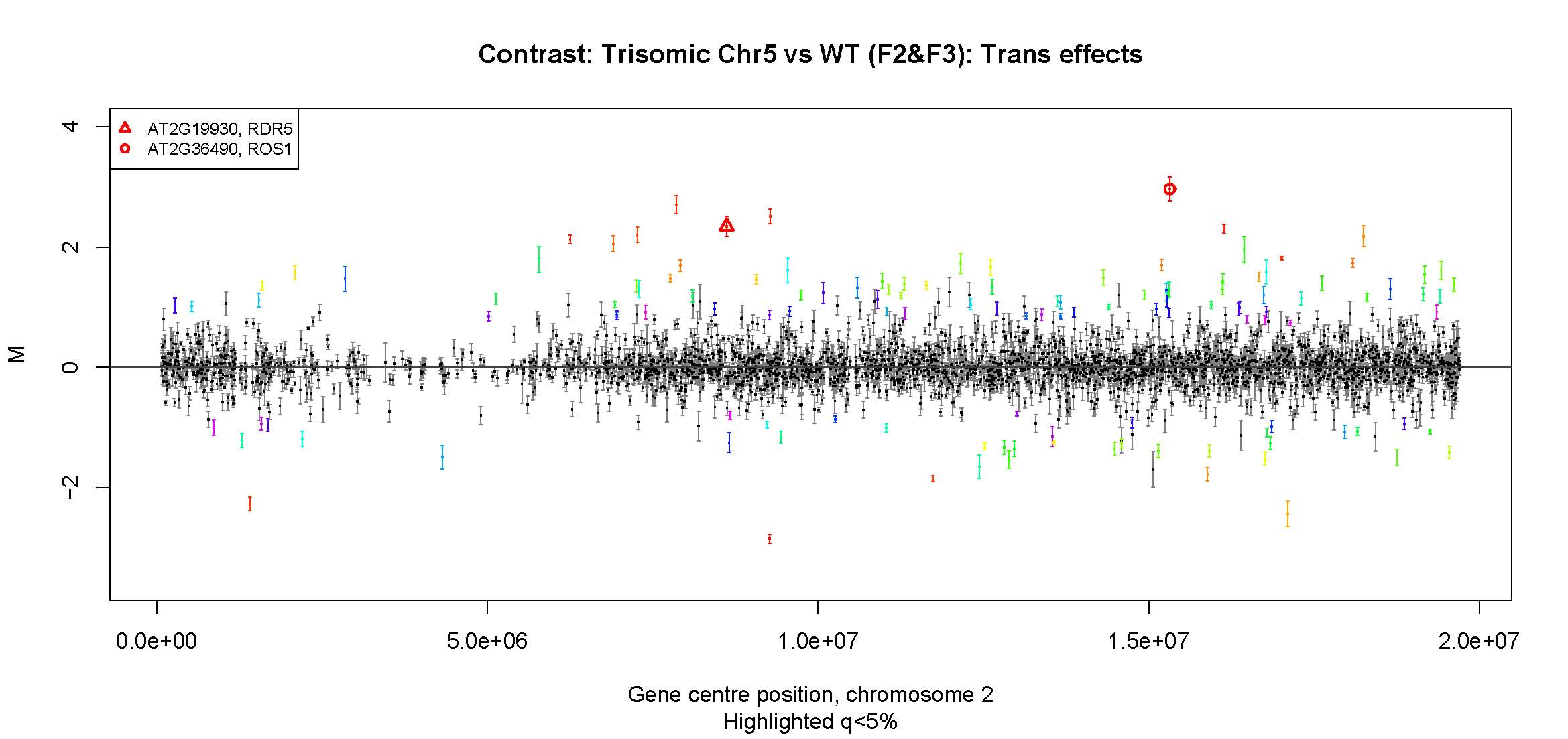


**Figure S4 - Huettel et al.**

Trans effects on the expression of genes on chromosome 2 in *Arabidopsis* plants trisomic for chromosome 5.

The panel shows the distribution of significant expression changes with transcripts represented by a mark and error bars. Highlighted are genes encoding the two epigenetic modifiers *ROS1* and *RDR5*, which are prominently up-regulated in chromosome 5 trisomic plants. The already highly expressed *ROS1* showed the 4^th^ strongest trans up-regulation (Holm FWER *p*_adj_<10^–5^), and *RDR5* was the 15^th^ most strongly up-regulated gene not on chromosome 5 (*p*_adj_<3×10^–5^). Both genes are among the 50 most significant trans effects (ranks 34 and 48). Full gene lists are provided in the Online Supplement. The *x*-axis corresponds to the gene centre locations along the chromosome, the *y*-axis shows expression change, with positive values signifying increased expression in the trisomic plants. Rainbow colours indicate relative significance (red/yellow is highest, blue/magenta is lowest).
