# Supplementary material for: Effects of Aneuploidy on Genome Structure, Expression, and Interphase Organization in Arabidopsis thaliana
Source: PLoS Genet. 2008 Oct 17;4(10):e1000226. doi: 10.1371/journal.pgen.1000226 (PMC2562519; doi:10.1371/journal.pgen.1000226)
Supplement: Figure S5 — Examples of connected YFP and DsRed dots for measurements of interallelic distances in three dimensions. (0.04 MB PDF) [file pgen.1000226.s005.pdf]

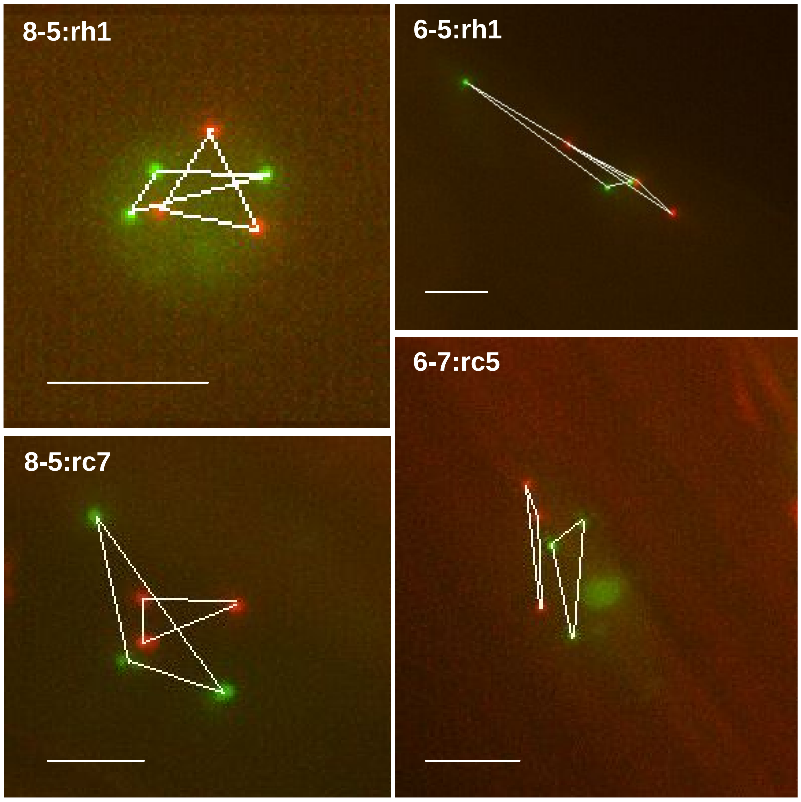

Figure S5. Huettel et al.
Examples of connected YFP and DsRed dots for measurements of inter-allelic distances and reconstructions in three-dimensions (Table S2).

The photographs show maximum projections (all stacks collapsed into one plane). The bar indicates 5 µm. The labelling denotes the plant tested. Triploids (8-5) are on the left; trisomics (6-5, 6-7) on the right.
