# Supplementary material for: Effects of Aneuploidy on Genome Structure, Expression, and Interphase Organization in Arabidopsis thaliana
Source: PLoS Genet. 2008 Oct 17;4(10):e1000226. doi: 10.1371/journal.pgen.1000226 (PMC2562519; doi:10.1371/journal.pgen.1000226)
Supplement: Figure S6 — Boxplot of normalized shortest interallelic distance. (0.03 MB DOC) [file pgen.1000226.s006.doc]

**Figure S6. Huettel et al.**

**Boxplot of normalized shortest distance between fluorescent-tagged sites on chromosome 5**.

The distance between three YFP and three DsRed fluorescent dots was normalized by selecting the proportion of the perimeter contributed by the smallest distance between dots (Table S2 A, B; column d3%). Thus, we account for a generally increased nuclear size in triploids compared to trisomics. For trisomics, the downward shift indicates a closer association between tagged sites as compared to triploids.
