# Supplementary material for: Effects of Aneuploidy on Genome Structure, Expression, and Interphase Organization in Arabidopsis thaliana
Source: PLoS Genet. 2008 Oct 17;4(10):e1000226. doi: 10.1371/journal.pgen.1000226 (PMC2562519; doi:10.1371/journal.pgen.1000226)
Supplement: Table S1 — List of plants. (0.18 MB DOC) [file pgen.1000226.s007.doc]

| **No.** | **ID** | **# nuclei** | **fluorescent** | **extra chr** | **predicted** | **actual # chr** | **deletion** | **transcriptome** |
| --- | --- | --- | --- | --- | --- | --- | --- | --- |
|  | **(F2)** |  | **tag** | **observed** | **# chr** | **(metaphase** | **(CGH)** | **(ATH1)** |
|  |  |  |  | **(CGH)** | **(CGH)** | **counting)** |  |  |
| **1** | **5-3** |  | **2R/2Y** | **None** | **10** | **10** | **No** |  |
| **2** | **5-5** |  | **2R/2Y** | **chr 1, 4** | **12** | **n.d.** | **No** |  |
| **3** | **5-7** |  | **2R/2Y** | **chr 1** | **11** | **n.d.** | **No** |  |
| **4** | **5-10** |  | **2R/2Y** | **chr 1** | **11** | **n.d.** | **No** |  |
| **5** | **6-1** |  | **3R/3Y** | **chr 2** | **16** | **16** | **No** |  |
| **6** | **6-4** |  | **2R/2Y** | **None** | **10** | **10** | **No** | **Yes** |
| **7** | **6-5** | **18** | **3R/3Y** | **chr 5** | **11** | **11** | **No** | **Yes** |
| **8** | **6-7** | **8** | **3R/3Y** | **chr 5** | **11** | **11** | **No** | **Yes** |
| **9** | **7-1** |  | **4R/4Y** | **chr 5** | **16** | **16** | **No** |  |
| **10** | **7-2** |  | **2R/2Y** | **None** | **10** | **10** | **No** | **Yes** |
| **11** | **7-3** |  | **3R/3Y** | **chr 2** | **16** | **16** | **No** |  |
| **12** | **7-5** |  | **3R/3Y** | **chr 2** | **16** | **16** | **No** |  |
| **13** | **7-6** |  | **4R/4Y** | **chr 5** | **16** | **16** | **No** |  |
| **14** | **7-7** |  | **2R/2Y** | **chr 1** | **n.d.** | **11** | **No** |  |
| **15** | **7-11** |  | **3R/3Y** | **chr 4, 5** | **12** | **12** | **No** |  |
| **16** | **8-1** |  | **4R/4Y** | **chr 2, 5** | **17** | **17** | **No** |  |
| **17** | **8-5** | **8** | **3R/3Y** | **None** | **15** | **15** | **No** |  |
| **18** | **8-6** | **4** | **3R/3Y** | **None** | **15** | **15** | **No** |  |
| **19** | **8-8** |  | **3R/3Y** | **chr 4, 5** | **12** | **12** | **No** |  |
| **20** | **8-9** |  | **2R/2Y** | **chr 2** | **11** | **11** | **No** |  |
| **21** | **8-10** |  | **2R/2Y** | **chr 2** | **11** | **11** | **No** |  |
| **22** | **8-11** |  | **3R/3Y** | **chr 2** | **16** | **16** | **No** |  |
| **23** | **9-1** | **10** | **3R/3Y** | **None** | **15** | **15** | **No** |  |
| **24** | **9-2** |  | **4R/4Y** | **None** | **20** | **20** | **No** |  |
| **25** | **9-5** |  | **3R/3Y** | **chr 4, 5** | **12** | **12** | **No** |  |
| **26** | **9-10** |  | **2R/2Y** | **chr 3** | **11** | **11** | **No** |  |
| **27** | **9-14** |  | **only 3Y** | **chr 2, 5** | **12** | **12** | **No** |  |
| **28** | **11-1** |  | **3R/3Y** | **chr 1, 2, 4, 5** | **14** | **14** | **No** |  |
| **29** | **11-2** |  | **3R/3Y** | **chr 2, 5** | **12** | **12** | **No** |  |
| **30** | **11-4** |  | **3R/3Y** | **chr 3** | **16** | **16** | **No** |  |
| **31** | **11-5** | **7** | **3R/3Y** | **None** | **15** | **15** | **del top arm chr 1, ca. 5.38 MB** |  |
| **32** | **11-7** |  | **2R/2Y** | **chr 2** | **11** | **11** | **No** |  |
| **33** | **11-10** |  | **2R/2Y** | **chr 4** | **11** | **11** | **No** |  |

**Table S1A – Huettel et al. List of plants**

The F2 progeny of five triploid plants (ID numbers in column 2) were inspected at the seedling stage by fluorescence microscopy and used for optical sectioning (number of nuclei for which stacks were made, column 3; results of interallelic distance measurements and 3D reconstructions in Tables S2A,B) and determining numbers of fluorescent dots (column 4). In the adult stage the plants were subjected to array CGH to determine unbalanced genomes and the identities of supernumerical chromosomes (columns 5 and 6). The actual chromosome number was validated by metaphase counting (column 7). In some cases chromosome-specific deletions were detected (column 8). Plants with a defined chromosome number (diploids or chromosome 5 trisomics) were selected for transcriptional profiling by microarray technology (column 9). Plants labelled in red letters are mentioned in the main text and figure legends.

| **No.** | **ID** | **# nuclei** | **fluorescent** | **extra chr** | **predicted** | **actual # chr** | **deletion** | **transcriptome** |
| --- | --- | --- | --- | --- | --- | --- | --- | --- |
|  | **(F3)** |  | **tag** | **observed** | **# chr** | **(metaphase** | **(CGH)** | **(ATH1)** |
|  |  |  |  | **(CGH)** | **(CGH)** | **counting)** |  |  |
| **1** | **6-5-1** |  | **3R/3Y or 4Y/4R** | **None** | **10** |  | **No** |  |
| **2** | **6-5-2** |  | **3R/3Y** | **chr 5** | **11** | **11** | **No** | **Yes** |
| **3** | **6-5-6** |  | **3R/3Y** | **chr 5** | **11** | **11** | **No** | **Yes** |
| **4** | **6-5-8** |  | **3R/3Y** | **chr 5** | **11** | **11** | **No** | **Yes** |
| **5** | **6-5-11** |  | **3R/3Y** | **chr 5** | **11** | **11** | **No** | **Yes** |
| **6** | **6-5-22** |  | **3R/2Y** | **chr 5** | **11** | **11** | **delta top arm chr 5** |  |
| **7** | **6-5-27** |  | **only 3R** | **chr 5** | **11** |  | **No** |  |
| **8** | **6-5-36** |  | **3R/3Y** | **chr 5** | **11** |  | **No** |  |
| **9** | **6-5-39** |  | **only 3R** | **chr 5** | **11** |  | **No** |  |
| **10** | **6-5-41** |  | **only 3R** | **chr 5** | **11** |  | **No** |  |
| **11** | **6-5-42** |  | **No signal** | **chr 5** | **11** |  | **No** |  |
| **12** | **6-5-47** |  | **3R/3Y** | **chr 5** | **11** | **11** | **No** |  |
| **13** | **6-5-48** |  | **only 3R** | **chr 5** | **11** | **11** | **No** |  |
| **14** | **6-7-4** |  | **3R/3Y** | **chr 5** | **11** |  | **No** |  |
| **15** | **6-7-6** |  | **3R/3Y** | **chr 5** | **11** |  | **No** |  |
| **16** | **6-7-9** |  | **3R/3Y** | **chr 5** | **11** |  | **No** |  |
| **17** | **6-7-10** |  | **3R/3Y** | **chr 5** | **11** | **11** | **delta bottom arm chr 5** |  |
| **18** | **6-7-19** |  | **3R/3Y** | **chr 5** | **11** | **11** | **No** | **Yes** |
| **19** | **6-7-20** |  | **3R/3Y** | **chr 5** | **11** | **11** | **No** | **Yes** |
| **20** | **6-7-21** |  | **3R/3Y** | **chr 5** | **11** | **11** | **No** | **Yes** |
| **21** | **6-7-22** |  | **3R/3Y** | **chr 5** | **11** | **11** | **No** | **Yes** |
| **22** | **6-7-23** |  | **3R/3Y** | **chr 5** | **11** |  | **No** |  |
| **23** | **6-7-53** |  | **3R/3Y** | **chr 5** | **11** | **11** | **No** |  |
| **24** | **6-7-57** |  | **only 3R** | **chr 5** | **11** |  | **No** |  |
| **25** | **6-7-58** |  | **3R/3Y** | **chr 5** | **11** |  | **No** |  |
| **26** | **6-7-59** |  | **3R/3Y** | **chr 5** | **11** |  | **No** |  |
| **27** | **6-7-62** |  | **only 3R** | **chr 5** | **11** |  | **No** |  |
| **28** | **6-4-1** |  | **2R/2Y** |  |  | **10** |  |  |
| **29** | **6-4-2** |  | **2R/2Y** |  |  | **10** |  |  |
| **30** | **6-4-3** |  | **2R/2Y** |  |  | **10** |  | **Yes** |
| **31** | **6-4-4** |  | **2R/2Y** |  |  | **10** |  | **Yes** |
| **32** | **7-2-1** |  | **2R/2Y** |  |  | **10** |  | **Yes** |
| **33** | **7-2-2** |  | **2R/2Y** |  |  | **10** |  |  |
| **34** | **7-2-3** |  | **2R/2Y** |  |  | **n.d.** |  |  |
| **35** | **7-2-4** |  | **2R/2Y** |  |  | **10** |  |  |

**Table S1B – Huettel et al.**

Legend as for part 1A except F3 plants in column 2 are progeny of trisomic (6-5, 6-7) or disomic (6-4, 7-2) F2 plants.
