# Supplementary material for: Effects of Aneuploidy on Genome Structure, Expression, and Interphase Organization in Arabidopsis thaliana
Source: PLoS Genet. 2008 Oct 17;4(10):e1000226. doi: 10.1371/journal.pgen.1000226 (PMC2562519; doi:10.1371/journal.pgen.1000226)
Supplement: Table S2 — Interallelic distance measurements. (0.36 MB DOC) [file pgen.1000226.s008.doc]

| **Triploid F2** |  |  |  |  |  |  |  |  |  |  |  |  |  |  |  |  |  |  |  |  |
| --- | --- | --- | --- | --- | --- | --- | --- | --- | --- | --- | --- | --- | --- | --- | --- | --- | --- | --- | --- | --- |
| **YFP** | **d1** | **d2** | **d3** | **tc** | **P** | **d1%** | **d2%** | **d3%** | **group** |  | **DsRed** | **d1** | **d2** | **d3** | **tc** | **P** | **d1%** | **d2%** | **d3%** | **group** |
| **YYY 8-5:rc1** | **9.44** | **5.53** | **4.20** | **B** | **19.10** | **49.24** | **28.85** | **21.91** | **II** |  | **RRR 8-5:rc1** | **8.16** | **7.44** | **2.43** | **B** | **18.03** | **45.26** | **41.26** | **13.48** | **I** |
| **YYY 8-5:rc5** | **15.92** | **9.76** | **6.74** | **B** | **32.42** | **49.11** | **30.10** | **20.79** | **II** |  | **RRR 8-5:rc5** | **20.05** | **11.46** | **8.96** | **B** | **40.47** | **49.54** | **28.32** | **22.14** | **II** |
| **YYY 8-5:rc6** | **3.83** | **2.15** | **1.97** | **B** | **7.95** | **48.18** | **27.04** | **24.78** | **II** |  | **RRR 8-5:rc6** | **5.62** | **3.68** | **2.53** | **B** | **11.83** | **47.11** | **31.11** | **21.39** | **II** |
| **YYY 8-5:rc7** | **11.39** | **7.53** | **5.89** | **E** | **24.81** | **45.91** | **30.35** | **23.74** | **II** |  | **RRR 8-5:rc7** | **5.57** | **5.08** | **3.75** | **C** | **14.40** | **38.68** | **35.28** | **26.04** | **II** |
| **YYY 8-5:rc8** | **4.59** | **4.13** | **3.65** | **E** | **12.37** | **37.11** | **33.39** | **29.51** | **II** |  | **RRR 8-5:rc8** | **4.27** | **4.24** | **1.50** | **C** | **10.01** | **42.66** | **42.36** | **14.99** | **I** |
| **YYY 8-5:rh1** | **4.40** | **3.42** | **1.48** | **E** | **9.30** | **47.31** | **36.77** | **15.91** | **I** |  | **RRR 8-5:rh1** | **4.95** | **3.96** | **3.50** | **D** | **12.41** | **39.89** | **31.91** | **28.20** | **II** |
| **YYY 8-5:rh2** | **2.53** | **2.30** | **1.76** | **C** | **6.59** | **38.39** | **34.90** | **26.71** | **II** |  | **RRR 8-5:rh2** | **11.03** | **7.55** | **5.20** | **B** | **23.78** | **46.88** | **31.75** | **21.87** | **II** |
| **YYY 8-5:rh3** | **11.72** | **8.72** | **3.09** | **B** | **23.53** | **49.81** | **37.06** | **13.13** | **I** |  | **RRR 8-5:rh3** | **2.21** | **1.81** | **1.08** | **C** | **5.10** | **43.33** | **35.49** | **21.18** | **II** |
| **YYY 8-6:rc2** | **7.11** | **4.74** | **4.67** | **D** | **16.52** | **43.04** | **28.69** | **28.27** | **II** |  | **RRR 8-6:rc2** | **9.29** | **6.78** | **2.79** | **B** | **18.86** | **49.26** | **35.95** | **14.79** | **I** |
| **YYY 8-6:rc5** | **11.71** | **10.63** | **4.92** | **C** | **27.26** | **42.96** | **38.99** | **18.05** | **I** |  | **RRR 8-6:rc5** | **7.83** | **6.83** | **5.69** | **E** | **20.35** | **38.48** | **33.56** | **27.96** | **II** |
| **YYY 8-6:rc6** | **16.67** | **11.74** | **6.05** | **B** | **34.46** | **48.37** | **34.07** | **17.56** | **I** |  | **RRR 8-6:rc6** | **14.93** | **8.43** | **7.05** | **B** | **30.41** | **49.10** | **27.72** | **23.18** | **II** |
| **YYY 8-6:rh1** | **4.20** | **3.55** | **2.62** | **C** | **10.37** | **40.50** | **34.23** | **25.27** | **II** |  | **RRR 8-6:rh1** | **4.02** | **3.40** | **3.11** | **D** | **10.53** | **38.18** | **32.29** | **29.53** | **II** |
| **YYY 9-1:rc1** | **11.80** | **8.61** | **3.76** | **B** | **24.17** | **48.82** | **35.62** | **15.56** | **I** |  | **RRR 9-1:rc1** | **5.78** | **3.74** | **3.32** | **D** | **12.84** | **45.02** | **29.13** | **25.86** | **II** |
| **YYY 9-1:rc2** | **8.40** | **6.86** | **3.50** | **E** | **18.76** | **44.78** | **36.57** | **18.66** | **II** |  | **RRR 9-1:rc2** | **11.11** | **7.99** | **3.18** | **B** | **22.28** | **49.87** | **35.86** | **14.27** | **I** |
| **YYY 9-1:rc3** | **9.54** | **8.54** | **1.22** | **B** | **19.30** | **49.43** | **44.25** | **6.32** | **I** |  | **RRR 9-1:rc3** | **14.01** | **8.42** | **6.50** | **B** | **28.93** | **48.43** | **29.10** | **22.47** | **II** |
| **YYY 9-1:rc4** | **9.46** | **9.21** | **6.36** | **C** | **25.03** | **37.79** | **36.80** | **25.41** | **II** |  | **RRR 9-1:rc4** | **6.62** | **3.97** | **3.30** | **B** | **13.89** | **47.66** | **28.58** | **23.76** | **II** |
| **YYY 9-1:rc5** |  |  |  |  |  |  |  |  |  |  | **RRR 9-1:rc5** | **8.75** | **5.90** | **3.87** | **B** | **18.52** | **47.25** | **31.86** | **20.90** | **II** |
| **YYY 9-1:rc6** |  |  |  |  |  |  |  |  |  |  | **RRR 9-1:rc6** | **14.19** | **9.04** | **5.24** | **B** | **28.47** | **49.84** | **31.75** | **18.41** | **I** |
| **YYY 9-1:rc7** | **11.71** | **8.42** | **3.68** | **B** | **23.81** | **49.18** | **35.36** | **15.46** | **I** |  | **RRR 9-1:rc7** |  |  |  |  |  |  |  |  |  |
| **YYY 9-1:rc8** | **12.74** | **6.81** | **6.24** | **D** | **25.79** | **49.40** | **26.41** | **24.20** | **II** |  | **RRR 9-1:rc8** |  |  |  |  |  |  |  |  |  |
| **YYY 9-1:rc9** | **13.03** | **9.17** | **5.76** | **E** | **27.96** | **46.60** | **32.80** | **20.60** | **II** |  | **RRR 9-1:rc9** | **16.49** | **13.87** | **2.78** | **B** | **33.14** | **49.76** | **41.85** | **8.39** | **I** |
| **YYY 9-1:rc10** | **5.69** | **4.24** | **2.41** | **E** | **12.34** | **46.11** | **34.36** | **19.53** | **II** |  | **RRR 9-1:rc10** | **3.40** | **3.16** | **2.36** | **C** | **8.92** | **38.12** | **35.43** | **26.46** | **II** |
| **YYY 11-5:rc1** | **8.36** | **6.31** | **4.59** | **E** | **19.26** | **43.41** | **32.76** | **23.83** | **II** |  | **RRR 11-5:rc1** | **9.87** | **8.45** | **3.71** | **C** | **22.03** | **44.80** | **38.36** | **16.84** | **I** |
| **YYY 11-5:rc5** | **9.72** | **7.38** | **2.87** | **B** | **19.97** | **48.67** | **36.96** | **14.37** | **I** |  | **RRR 11-5:rc5** | **4.16** | **2.30** | **2.17** | **D** | **8.63** | **48.20** | **26.65** | **25.15** | **II** |
| **YYY 11-5:rc8** | **8.60** | **8.35** | **1.92** | **C** | **18.87** | **45.57** | **44.25** | **10.18** | **I** |  | **RRR 11-5:rc8** | **8.59** | **8.45** | **3.25** | **C** | **20.29** | **42.33** | **41.65** | **16.02** | **I** |
| **YYY 11-5:rc9** | **14.65** | **13.93** | **2.71** | **C** | **31.29** | **46.82** | **44.52** | **8.66** | **I** |  | **RRR 11-5:rc9** | **13.35** | **9.42** | **4.64** | **B** | **27.41** | **48.70** | **34.37** | **16.93** | **I** |
| **YYY 11-5:rh2** | **8.71** | **8.16** | **5.25** | **C** | **22.12** | **39.38** | **36.89** | **23.73** | **II** |  | **RRR 11-5:rh2** | **9.09** | **7.07** | **5.25** | **E** | **21.41** | **42.46** | **33.02** | **24.52** | **II** |
| **YYY 11-5:rh4** | **5.63** | **4.96** | **2.45** | **E** | **13.04** | **43.17** | **38.04** | **18.79** | **II** |  | **RRR 11-5:rh4** | **4.17** | **3.43** | **2.78** | **E** | **10.38** | **40.17** | **33.05** | **26.78** | **II** |
| **YYY 11-5:rh6** | **2.34** | **2.02** | **1.10** | **C** | **5.46** | **42.86** | **36.99** | **20.15** | **II** |  | **RRR 11-5:rh6** | **2.48** | **1.72** | **1.59** | **D** | **5.79** | **42.83** | **29.71** | **27.46** | **II** |
| **Average** | **9.03** | **6.93** | **3.74** | **A: 0** | **19.70** | **45.26** | **35.07** | **19.67** | **I: 10** |  |  | **8.52** | **6.21** | **3.76** | **A: 0** | **18.49** | **44.96** | **33.61** | **21.44** | **I: 9** |
|  |  |  |  | **B: 7** |  |  |  |  | **II: 17** |  |  |  |  |  | **B:12** |  |  |  |  | **II: 18** |
|  |  |  |  | **C: 9** |  |  |  |  |  |  |  |  |  |  | **C: 5** |  |  |  |  |  |
|  |  |  |  | **D: 1** |  |  |  |  |  |  |  |  |  |  | **D: 5** |  |  |  |  |  |
|  |  |  |  | **E: 7** |  |  |  |  |  |  |  |  |  |  | **E: 3** |  |  |  |  |  |

**Table S2A – Huettel et al. Interallelic distance measurements in triploids (legend and abbreviations below)**

| **Trisomic F2** |  |  |  |  |  |  |  |  |  |  |  |  |  |  |  |  |  |  |  |  |
| --- | --- | --- | --- | --- | --- | --- | --- | --- | --- | --- | --- | --- | --- | --- | --- | --- | --- | --- | --- | --- |
| **YFP** | **d1** | **d2** | **d3** | **tc** | **P** | **d1%** | **d2%** | **d3%** | **group** |  | **DsRed** | **d1** | **d2** | **d3** | **tc** | **P** | **d1%** | **d2%** | **d3%** | **group** |
| **YYY 6-5:rc1** | **4.82** | **4.39** | **1.78** | **C** | **10.99** | **43.86** | **39.95** | **16.20** | **I** |  | **RRR 6-5:rc1** | **7.68** | **6.21** | **3.41** | **C** | **17.30** | **44.39** | **35.90** | **19.71** | **II** |
| **YYY 6-5:rc3** | **3.07** | **2.78** | **0.73** | **C** | **6.58** | **46.66** | **42.25** | **11.09** | **I** |  | **RRR 6-5:rc3** | **1.83** | **1.17** | **0.29** | **E** | **2.79** | **47.67** | **41.94** | **10.39** | **I** |
| **YYY 6-5:rc4** | **5.72** | **4.21** | **1.75** | **B** | **11.68** | **48.97** | **36.04** | **14.98** | **I** |  | **RRR 6-5:rc4** | **7.23** | **5.64** | **2.24** | **E** | **15.11** | **47.85** | **37.33** | **14.82** | **I** |
| **YYY 6-5:rc5** |  |  |  |  |  |  |  |  |  |  | **RRR 6-5:rc5** | **5.30** | **4.40** | **3.66** | **E** | **13.36** | **39.67** | **32.93** | **27.40** | **II** |
| **YYY 6-5:rc6** | **3.29** | **2.50** | **1.46** | **E** | **7.25** | **45.38** | **34.48** | **20.14** | **II** |  | **RRR 6-5:rc6** |  |  |  |  |  |  |  |  |  |
| **YYY 6-5:rc7** | **4.70** | **3.67** | **2.29** | **E** | **10.66** | **44.09** | **34.43** | **21.48** | **II** |  | **RRR 6-5:rc7** |  |  |  |  |  |  |  |  |  |
| **YYY 6-5:rc8** | **3.01** | **2.46** | **1.88** | **C** | **7.35** | **40.95** | **33.47** | **25.58** | **II** |  | **RRR 6-5:rc8** |  |  |  |  |  |  |  |  |  |
| **YYY 6-5:rc9** | **2.74** | **2.65** | **2.02** | **C** | **7.41** | **36.98** | **35.76** | **27.26** | **II** |  | **RRR 6-5:rc9** |  |  |  |  |  |  |  |  |  |
| **YYY 6-5:rc11** | **3.94** | **2.48** | **1.60** | **E** | **8.02** | **49.13** | **30.92** | **19.95** | **II** |  | **RRR 6-5:rc11** |  |  |  |  |  |  |  |  |  |
| **YYY 6-5:rc12** | **2.88** | **1.64** | **1.33** | **D** | **5.85** | **49.23** | **28.08** | **22.74** | **II** |  | **RRR 6-5:rc12** |  |  |  |  |  |  |  |  |  |
| **YYY 6-5:rc13** | **8.90** | **7.08** | **4.29** | **C** | **20.27** | **43.91** | **34.93** | **21.16** | **II** |  | **RRR 6-5:rc13** |  |  |  |  |  |  |  |  |  |
| **YYY 6-5:rc15** | **4.01** | **3.71** | **2.57** | **C** | **10.29** | **38.97** | **36.05** | **24.98** | **II** |  | **RRR 6-5:rc15** |  |  |  |  |  |  |  |  |  |
| **YYY 6-5:rc16** | **4.79** | **4.66** | **0.58** | **C** | **10.03** | **47.76** | **46.46** | **5.78** | **I** |  | **RRR 6-5:rc16** |  |  |  |  |  |  |  |  |  |
| **YYY 6-5:rc17** | **3.17** | **2.05** | **1.68** | **D** | **6.90** | **45.94** | **29.71** | **24.35** | **II** |  | **RRR 6-5:rc17** |  |  |  |  |  |  |  |  |  |
| **YYY 6-5:rc18** | **7.30** | **4.81** | **2.70** | **B** | **14.81** | **49.29** | **32.48** | **18.23** | **I** |  | **RRR 6-5:rc18** | **6.15** | **5.08** | **2.94** | **C** | **14.17** | **43.40** | **35.85** | **20.75** | **II** |
| **YYY 6-5:rc19** |  |  |  |  |  |  |  |  |  |  | **RRR 6-5:rc19** | **7.94** | **7.60** | **0.46** | **C** | **16.00** | **49.63** | **47.50** | **2.88** | **I** |
| **YYY 6-5:rh1** | **14.31** | **13.21** | **1.97** | **C** | **29.49** | **48.52** | **44.79** | **6.68** | **I** |  | **RRR 6-5:rh1** | **9.35** | **5.97** | **3.56** | **E** | **18.88** | **49.52** | **31.62** | **18.86** | **II** |
| **YYY 6-5:rh3** | **4.08** | **3.41** | **1.25** | **C** | **8.74** | **46.68** | **39.02** | **14.30** | **I** |  | **RRR 6-5:rh3** |  |  |  |  |  |  |  |  |  |
| **YYY 6-7:rc1** | **4.48** | **3.69** | **1.79** | **C** | **9.96** | **44.98** | **37.05** | **17.97** | **I** |  | **RRR 6-7:rc1** | **7.76** | **4.48** | **3.48** | **E** | **15.72** | **49.36** | **28.50** | **22.14** | **II** |
| **YYY 6-7:rc3** | **7.75** | **5.70** | **4.15** | **E** | **17.60** | **44.03** | **32.39** | **23.58** | **II** |  | **RRR 6-7:rc3** | **4.15** | **3.24** | **3.01** | **D** | **10.40** | **39.90** | **31.15** | **28.94** | **II** |
| **YYY 6-7:rc4** | **8.11** | **5.57** | **3.03** | **E** | **16.71** | **48.53** | **33.33** | **18.13** | **I** |  | **RRR 6-7:rc4** | **7.69** | **5.82** | **2.42** | **E** | **15.93** | **48.27** | **36.53** | **15.19** | **I** |
| **YYY 6-7:rc5** | **6.24** | **5.46** | **1.97** | **C** | **13.67** | **45.65** | **39.94** | **14.41** | **I** |  | **RRR 6-7:rc5** | **6.34** | **4.70** | **1.92** | **E** | **12.96** | **48.92** | **36.27** | **14.81** | **I** |
| **YYY 6-7:rc8** | **5.94** | **4.54** | **2.27** | **E** | **12.75** | **46.59** | **35.61** | **17.80** | **I** |  | **RRR 6-7:rc8** | **2.72** | **2.56** | **2.14** | **A** | **7.42** | **36.66** | **34.50** | **28.84** | **II** |
| **YYY 6-7:rc9** | **4.38** | **3.19** | **1.54** | **B** | **9.11** | **48.08** | **35.02** | **16.90** | **I** |  | **RRR 6-7:rc9** | **12.38** | **8.25** | **4.67** | **E** | **25.30** | **48.93** | **32.61** | **18.46** | **I** |
| **YYY 6-7:rc10** | **9.97** | **8.02** | **2.26** | **C** | **20.25** | **49.23** | **39.60** | **11.16** | **I** |  | **RRR 6-7:rc10** | **8.31** | **7.98** | **0.62** | **C** | **16.91** | **49.14** | **47.19** | **3.67** | **I** |
| **YYY 6-7:rc11** | **9.32** | **6.13** | **5.04** | **D** | **20.49** | **45.49** | **29.92** | **24.60** | **II** |  | **RRR 6-7:rc11** | **3.59** | **3.38** | **1.45** | **C** | **8.42** | **42.64** | **40.14** | **17.22** | **I** |
| **Average** | **5.71** | **4.50** | **2.16** | **A: 0** | **12.37** | **45.79** | **35.90** | **18.31** | **I: 13** |  |  | **6.56** | **5.10** | **2.42** | **A: 1** | **14.04** | **45.73** | **36.66** | **17.61** | **I: 8** |
|  |  |  |  | **B: 3** |  |  |  |  | **II: 11** |  |  |  |  |  | **B: 0** |  |  |  |  | **II: 7** |
|  |  |  |  | **C:12** |  |  |  |  |  |  |  |  |  |  | **C: 4** |  |  |  |  |  |
|  |  |  |  | **D: 3** |  |  |  |  |  |  |  |  |  |  | **D: 1** |  |  |  |  |  |
|  |  |  |  | **E: 6** |  |  |  |  |  |  |  |  |  |  | **E: 8** |  |  |  |  |  |

**Table S2B – Huettel et al. Interallelic distance measurements in trisomics**

**Table S2: Distances between fluorescent-tagged sites on chromosome 5 in living triploid (2A) and trisomic (2B) F2 seedlings.**

Three distance measurements in micrometers (d1, d2, d3) were made between YFP (Y) and DsRed (R) signals in individual root interphase nuclei (leftmost columns) in the two trisomic F2 plants (6-5 and 6-7) and four triploid F2 plants (8-5, 8-6, 9-1,11-5). 3D reconstructions of nuclei were made to determine triangle categories (examples of maximum projections in Figure S5).

Empty spaces indicate that no fluorescence dots were visible, which was presumably due to silencing of either the *TetR-YFP* or *DsRed-LacI* transgene. Silencing was more pronounced in trisomic than in triploid seedlings and was even exacerbated in the F3 generation (not shown).

The group I to group II ratio was approximately 2:1 for triploids and 1:1 for trisomics (red numbering). In other words, two transgene alleles were more often closely apposed (shortest ‘d’ < 18.5% of perimeter) in trisomics than in triploids. These results are depicted in a box plot in Figure S6. Inter-allelic distances of ~ 0.5 µm, suggesting pairing, were detected in some trisomics (highlighted yellow).

**Abbreviations:**

d = distances in µm

tc = triangle categories, 2 distances (d) are considered approximately the same (~) if the lower value is greater than or equal to 80% of the higher value A: d1~d2~d3 - equilateral; B: d1~d2+d3; C: d1~d2>d3 - isosceles; D: d1>d2~d3 - isosceles; E: d1>d2>d3 – scalene

p (perimeter) = sum of distances d1+d2+d3

d% = each distance as percent of perimeter

groups - shortest distance 0 to 18.49%: I; shortest distance 18.50 to 33.33%: II

rc = root cell; rh = root hair
