# Supplementary material for: Effects of Aneuploidy on Genome Structure, Expression, and Interphase Organization in Arabidopsis thaliana
Source: PLoS Genet. 2008 Oct 17;4(10):e1000226. doi: 10.1371/journal.pgen.1000226 (PMC2562519; doi:10.1371/journal.pgen.1000226)
Supplement: Table S3 — Primers. (0.03 MB DOC) [file pgen.1000226.s009.doc]

**Table S3 – Huettel et al.**

Primers for qRT-PCR

Transgene 1 YFP CGACAACCACTACCTGAGCTACCA

GAACTCCAGCAGGACCATGTGAT

Transgene 2 LacI GTTATATCCCGCCGTCAACCAC

CAACAGCTGATTGCCCTTCAC

__________________________________________________________

AT2G19930 RDR5 TGACACAGGCGATGAAGACTGAT

CGCCATAGAACTCCTG^CTTGTATCT

AT2G36490 ROS1 CCTCTGTTCCTACGATATTCAAAGGT

TTCGATCAAATCCACGTACACATAC

AT5G10140 FLC GCTTCAACATGAGTTCGGT^CTTC

AAGCTTGTGGGATCAAATGTCA

AT5G54670 ATK3 CACATATCTTCTCCAG^CCTTGC

GAAGAAGATTCCGGTGCAATGT

AT5G62040 BFT ATATGCGTGAATATCTGCATT^GA

CCGGTTTAGGCGTCTCATATCT

AT5G65070 MAF4 TGAAGACCCATCAAGAGAAG^GA

ATCCTCTGCTTCCACAGACTTCTT

AT5G39630 AAACAGCCAAGCTATG^CTTCA

TTTATGCTTCTCACGATTGACC

AT5G57720 TGCCGTCAAAGTTTAAGCCATA

TTCCAGGCGTATATTGGTAAT^TG

AT5G47635 TTAATCCATTTGGACCTCCTTTT

ACTTCACGTCCTCACCTTCACT

AT5G13380 TCTTGTCACAACGTTTTCTGGT^C

ACATTCTGTCTCCCCACGAAGT

AT5G40100 ACTGAGCCTCCAGTTTCTCGAC

TGTGGTTGTGCTGATACCT^CTC

At5g24900 CYP714A2 ATGCTTCTCTTCCCACGATTG

TCCTAATCTCAAAACGGT^GA

AT5G55520 ATGCCGAAACGTTCCT^CAG

TCAAGCTGCTGGAGAATTA

__________________________________________________________

AT1G13320 PP2A TAACGTGGCCAAAATGATGC

GTTCTCCACAAC^CGCTTGGT

AT1G13440 GAPC-2 TTGGTGACAACAG^GTCAAGCA

AAACTTGTCGCTCAATGCAATC

AT3G53090 UPL7 TACGTGCTACAATACTCTTAAG^CTTCC

TTTTCCCTCATAGTGCTTGCTC

AT5G25760 PEX4 CTGCGACTCAG^GGAATCTTCTAA

TTGTGCCATTGAATTGAACCC

Note: "^" denotes an exon/intron junction

Primer sequence information for reference genes AT1G13440, AT1G13320, AT5G25760 was taken from Czechowski *et al.* (2005).

Czechowski T, Stitt M, Altmann T, Udvardi MK, Scheible WR (2005) Genome-wide identification and testing of superior reference genes for transcript normalization in *Arabidopsis*. Plant Physiol 139:5-17.
