# Supplementary material for: Effects of Aneuploidy on Genome Structure, Expression, and Interphase Organization in Arabidopsis thaliana
Source: PLoS Genet. 2008 Oct 17;4(10):e1000226. doi: 10.1371/journal.pgen.1000226 (PMC2562519; doi:10.1371/journal.pgen.1000226)
Supplement: Text S1 — Supporting information text. (0.27 MB PDF) [file pgen.1000226.s010.pdf]

Supporting Material:

Text of the Online Supplement archived at

http://bioinf.boku.ac.at/pub/trisomy2008

For convenience, these pages also include the text of the Online Supplement albeit without figures, whereas the Online Supplement provides many complementary figures and tables fully linked from the online text.

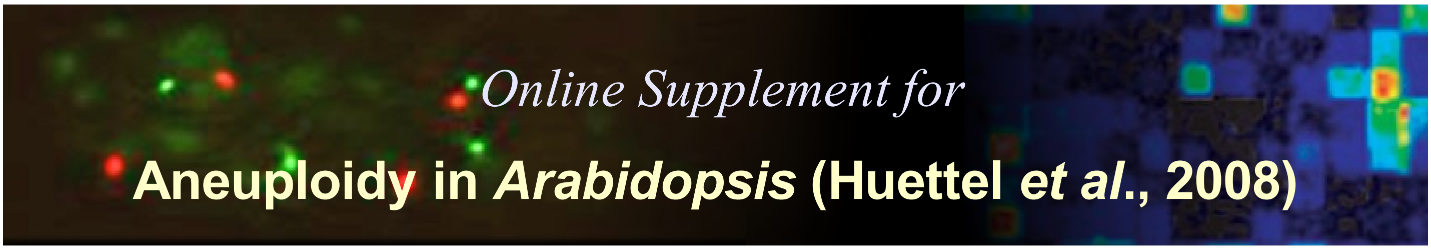

Effects of Aneuploidy on Genome Structure, Expression and Interphase Organization in *Arabidopsis thaliana*

Bruno Huettel,[1] David P. Kreil,[1] Marjori Matzke,[*] and Antonius J. M. Matzke

These pages provide supplementary information to the journal article [submitted to | published in]

PLoS Genetics **vol**, pages.

Microarray data has been submitted to the public ArrayExpress repository (accession number EMEXP-1454, pre-publication access requires reviewer password).

Supplementary figures and tables explicitly referenced in the manuscript are provided in section *Microarray Results*.

Section *Microarray Methods* contains diagnostic plots for the low-level array data analysis and transforms and discusses normalization choices.

Finally, the *Fluorescence Microscopy* section provides electronic copies of the fluorescence microscopy photographs shown in the paper. [***Photos in Online Supplement only***]

Microarray Methods

Robust multi-chip probe-level model diagnostic plots

This section provides the fully annotated expression data table after low-level transforms and normalization, and low-level analysis diagnostic plots.

After reannotation (v10 for TAIR v7; Dai *et al.*, 2005) and filtering of probe sets probe sequence specific effects were removed (Wu *et al.*, 2004) as described in our manuscript's Method section. Then probe level signals were normalized for different backgrounds and overall hybridization intensities of individual chips using an iterative 20%-trimmed least squares fit of a generative model with additive-multiplicative noise (Huber *et al.*, 2002) using a $\log_2$-asymptotic calibration, and excluding chromosome 5 genes from the parameter fitting step. Finally, transcript expression estimates were obtained by robust fits of linear multi-chip probe level intensity models (Bolstad, 2004).

We here provide the full results and diagnostic plots for the fit:

- Fully annotated results [7.7 MB .dat.bz] are provided in bzip2 compressed format. The file contains a TAB-delimited text table that can be loaded in any spreadsheet program after decompression. Columns with a header starting with `expr.` contain the expression level estimates, and those with a header starting with `se.expr.` their standard errors. The remaining columns provide annotation compiled from TAIR (v7) and Affymetrix probe information.
- An uneven spatial distribution of fit residuals [38.9 MB PDF] or robust fit weights [34.3 MB PDF] can indicate process problems. No such trends, however, were observed.
- All *vs* all Q–Q / scatter plots [31.2 MB PDF] and *M(A)* plots [20.8 MB PDF] indicated that conditions for further normalization steps using popular approaches like *M(A)*-Loess or quantile–quantile normalization were not satisfied. This is seen even more clearly in Q–Q / *M(A)* plot for the group-wise averages [1.1 MB PDF], which are complemented by within-group comparisons [F0.WT | F1.WT | F0.tri5 | F1.tri5],

In the above files, for historical reasons, samples from the F2 and F3 generations were labelled F0 and F1, respectively.

Discussion of normalization transforms

We here first demonstrate the effect of applying to our data two commonly used normalization transforms and contrast this to our conservative approach. We then briefly consider the question of housekeeping genes in this context. Finally, we examine the interesting idea of using CGH measurements for signal normalization.

Choosing of an appropriate normalization transform (Kreil & Russell, 2005) needs to consider

- The assumptions on which the normalization transform rests.
  For example, normalization *via* division by the median signal per sample assumes that the median expression level of different samples should be the same. In our situation, where we expect many genes on the trisomic chromosome 5 to be expressed at higher levels in trisomic plants than in disomic plants, this requirement is not satisfied.
- The aggressiveness of the normalization transform.
  One distinguishes conservative transforms, which have very few parameters, and aggressive transforms which are more powerful but also prone to overfitting. Overfitting in this context means that the transform may consider true data features as technical bias. Removing the wrongly estimated technical trend then not only subtracts biological signal but may, moreover, introduce signal artefacts. In general, one therefore aims for a transform as conservative as possible yet powerful enough to remove technical artefacts in array measurements, such as labelling yields differing between samples.

Quantile normalization is a very generic and powerful normalization transform. As its underlying assumption, that the signal distribution for all samples should be the same, is valid in many experiments, it is popular and the default normalization method for many algorithms, such as the `rma` and `gcrma` approaches to probe summarization (Irizarry *et al.*, 2003; Wu *et al.*, 2004; Wu & Irizarry, 2005), which are provided in both academic and commercial tools (*e.g.*, Bioconductor or GeneSpring). In our situation, however, where we expect many genes on the trisomic chromosome 5 to be expressed at higher levels in trisomic plants than in disomic plants, the signal distribution in trisomic plants will reflect the large number of more highly expressed genes. Note that this is not easily seen from the diagnostic Q–Q plots above but follows directly from the large imbalance of differential signals shown in the *M(A)* plots. These indicate that a large number of genes are more highly expressed in the trisomic samples whereas no correspondingly strong down-regulation effect can be observed. As a result, the conditions for quantile normalization are not satisfied.

If quantile normalization is nevertheless applied, we observe a substantial squashing of the systematically increased expression of genes on chromosome 5. Consider the *M(A)* plots for the group-wise averages. Compare, *e.g.*, the third row of panels examining expression levels in wildtype (F1.WT) and trisomic plants (F1.tri5) of the F3 generation [conservative *vs* quantile normalization].

We think that this resolves the puzzle that some similar studies (*e.g.,* Makarevitch *et al.*, 2008) could detect only a fraction of the effects that we report in our paper. To test this assumption, we have tried to recreate the analysis reported there. To remove any unfair advantages of a better signal-to-noise ratio in larger data sets, we selected a subset of our data for comparable sample sizes. We then applied quantile normalization and used the statistical methods and thresholds of Makarevitch *et al.* (2008) in testing for differential expression (Benjamini-Hochberg correction for multiple testing, 15% FDR threshold). *Trans* effects almost vanished (7 genes affected), and for

only 23% of genes on chromosome 5 could higher expression levels be detected with significance. Restricting the analysis to more highly expressed genes, where the platform sensitivity is better, the proportion of affected *cis* genes rises to 44%, which is matches the numbers reported by Makarevitch *et al.* (2008). It is noteworthy that this is still a factor of two below the ratio of genes clearly affected in our study despite us using much more conservative statistical tests and thresholds. This difference is not due to greater sample size in our study, as we can recover most effects on chromosome 5 already with the smaller test set examined above. We conclude that inappropriate normalization can drastically affect the power of an analysis to detect even the large-scale expression differences caused by aneuploidy. This raises the hope that a re-analysis of earlier experiments may yield a considerably more sensitive detection of effects.

***M(A)* Loess normalization** is a another generic and powerful normalization transform. As its underlying assumption, that the differential expression signal should be independent of average gene expression levels and average to zero, is valid in many experiments, it is also popular and the default normalization method in many academic and commercial tools (*e.g.*, Bioconductor or Agilent Feature Extraction Software).

In our situation, however, where we expect many genes on the trisomic chromosome 5 to be expressed at higher levels in trisomic plants than in disomic plants, we do not expect differential expression signals to average to zero. This follows directly from the large imbalance of differential signals seen in the *M(A)* plots. These indicate that a large number of genes are more highly expressed in the trisomic samples whereas no correspondingly strong down-regulation effect can be observed. As a result, the conditions for *M(A)* Loess normalization normalization are not satisfied.

While this problem has been recognized (Torres *et al.*, 2007), there is no easy *post hoc* fix once an aggressive normalization transform has been applied. We illustrate this following the approach suggested by Torres *et al.* (2007). To this end, we have marked the *M(A)* Loess average by a white line in an *M(A)* plot similar to Figure 5 of our paper [pre.loess]. In Loess normalization, this average is subtracted from the signal *M* for all genes. The orange lines indicate the average expression and standard deviations for genes not on the trisomic chromosome 5, purple lines show the trend and variance for chromosome 5. It is clear that the orange centre trend line **before** Loess normalization shows the desired behaviour, hugging the zero axis. Subtracting the white general trend, in contrast, yielded data that then claimed that, on average, genes on other chromosomes showed reduced expression in trisomics. To fix this, Torres *et al.* (2007) suggested correcting data by the average *M* value of these genes after Loess normalization, the value of which is indicated by a yellow dashed line. Results of *M(A)* Loess normalization and this average bias subtraction fix, however, are worse than before normalization [post.loess]: The trend of genes on other chromosomes clearly deviates from the zero axis, showing a bias for upregulation for lowly and highly expression genes and also a bias for downregulation for genes with moderate expression levels.

A traditional approach to microarray normalization tries to exploit so-called **housekeeping genes**, assuming that such genes can be identified that indeed do not change under experimental conditions. Clearly, a gene that is stable in many conditions might still be affected by a particular experiment. It is hence interesting to briefly consider whether traditional housekeeping genes are stably expressed in a setting like ours where substantial non-random gene expression changes are expected. We therefore compared, for all samples, the measurements probing ribosomal RNA and actin expression levels as well as measurements of two other traditional house keeping genes, GAPDH (GAPC-2) and UPL7 [house]. One of the two RNA species, several actin genes, as well as GAPC-2 and UPL7 showed stable expression levels and thus also corroborate our normalization approach. Array measurements furthermore agreed well with qRT-PCR measurements [Fig. S1, bottom panel shows GAPC-2]. Interestingly, however, other supposedly stable genes like 25S rRNA and actin 11 showed marked fluctuations. This confirms that the selection of a good set of housekeeping genes is in general non-trivial. Normalization with a small number of housekeeping genes, moreover, suffers considerable noise compared to normalization methods that estimate their parameters from thousands of genes.

In comparison, in our **conservative approach** we identify, by iterative trimmed least squares fit, the subset of 60% least varying genes across all samples, and use these to identify a constant background offset and a constant multiplicative scaling factor. Only two parameters per sample need to be estimated, which makes the approach very conservative. As the parameter estimates are based on expression levels measured for thousands of genes they are, moreover, very precise. An established implementation of this method is available in the `R` package `vsn` (Huber *et al.*, 2002). We have further excluded all genes on chromosome 5 to ensure that the systemic overexpression of these genes did not affect the normalization procedure. Results with and without genes on chromosome 5 were very similar (data not shown), confirming the expected robustness of the approach to outliers. It is remarkable that this conservative transform already yielded highly consistent data, as is particularly reflected by the centre trend for genes not on chromosome 5 hugging the zero axis (orange lines of Figure 5 of the paper and the Supplement Results section).

As CGH data was available for a number of samples, we could also investigate the performance of using this data for normalization. This approach assumes that the fold-change in expression observed for each gene should reflect the fold-change in dosage measured by CGH. One might hope that gene specific differences in binding behaviour would similarly affect gene transcripts and genomic DNA targets. Interestingly, while the approach worked reasonably well for genes of moderate expression levels (slightly undercorrecting for gene dosage), considerable overcorrection was observed for low and high expression levels [CGH.norm]. These difficulties can be explained by the strong non-linear signal response of the platform that is also reflected in Figure 5 of the manuscript.

References

1. Bolstad B (2004). Low Level Analysis of High-density Oligonucleotide Array Data: Background, Normalization and Summarization. *Dissertation*. University of California, Berkeley, U.S.A. [reprint]
2. Huber W, von Heydebreck A, Sültmann H, Poustka A, Vingron M. (2002) Variance stabilization applied to microarray data calibration and to the quantification of differential expression. *Bioinformatics* **18**, S96. PMID 12169536
3. Irizarry RA, Bolstad BM, Collin F, Cope LM, Hobbs B, and Speed TP (2003). Summaries of Affymetrix GeneChip probe level data. *Nucleic Acids Res.* **31**, e15. PMID 12582260
4. Kreil DP and Russell RR (2005). There is no silver bullet – a guide to low-level data transforms and normalisation methods for microarray data. *Brief. Bioinf.* **6**, 86. PMID 15826359
5. Makarevitch I, Phillips RL, and Springer NM (2008). Profiling expression changes caused by a segmental aneuploid in maize. *BMC Genomics* **9**, 7. PMID 18186930
6. Torres EM, Sokolsky T, Tucker CM, Chan LY, Boselli M, Dunham MJ, and Amon A (2007). Effects of Aneuploidy on Cellular Physiology and Cell Division in Haploid Yeast. *Science* **317**, 916. PMID 17702937
7. Wu Z, Irizarry RA, Gentleman R, Martinez Murillo F, and Spencer F (2004). A model based background adjustment for oligonucleotide expression arrays. J. Am. Stat. Assoc. 99, 909.
8. Wu Z and Irizarry RA (2005). A Statistical Framework for the Analysis of Microarray Probe-Level Data. Johns Hopkins University, Biostatistics Working Papers 73.

Microarray Results

This section provides figures illustrating the observed expression changes (both trends and deviations from the trends), links to fully annotated tables of affected genes, and tables showing trends by GOslim category. Please scroll down for Figures S3 and S4.

Expression Changes – Figures

Figure 5 of our manuscript shows the systemic increase of expression for chromosome 5 genes in the trisomic plants, as a function of the average gene expression level (on the *x*-axis). Transcripts on chromosome 5 are coloured green, and the intensity dependent trend plus/minus standard deviation is plotted in magenta. The trend for transcripts on other chromosomes is shown in orange. The dashed vertical line marks the intensity A_{1+1} where the lower magenta and the upper orange lines cross and the trends are separated by 1+1 standard deviations. Surveys of trends thus focus on the strongly expressed transcripts to the right of the dashed lines, where the assay will be most accurate (*). The dotted vertical line indicates the lowest expression intensity for which a statistically significant change could be detected with $p < 5\%$ (Holm FWER). We here provide the figure in different resolutions [low | medium | high].

(*) The improved separation of more strongly expressed chromosome 5 genes from the trend of genes on other chromosomes that is evident in Figure 5 of our manuscript affects our ability to detect expression differences between trisomic and disomic samples. By plotting the percentage of genes on chromosome 5 that could be identified as having higher expression in trisomics, we can show that the sensitivity of our assay improves considerably for genes with an average intensity larger than approximately A_{1+1} and that the exact choice of the threshold makes little difference [Athresh]. To test the dosage-related increase in expression for genes on the trisomic chromosome 5 with lower expression levels, four moderately and five lowly expressed genes were selected for examination by high-sensitiviy qRT-PCR. Since the quantification of lowly expressed genes by real-time PCR can be non-trivial, genes were chosen with a well-documented exon/intron structure according to TAIR. The primers were designed to span the exon/intron junctions in order to ensure that PCR amplification did in fact report on differential gene expression rate and not, *e.g.*, on residual genomic DNA. Consistent with the general chromosome 5 trend, a higher steady state transcript level in trisomics was indeed observed for the majority of these genes [Fig. S1 and Fig. S2].

Figure 4 of our manuscript shows that all areas of the triplicated chromosome seem similarly affected [cis.Mpos] and that the observed trans effects appear to scatter randomly across chromosomes [trans.Mpos]. In these plots only strongly expressed genes, for which the effect could be assessed accurately are shown.

For a study of deviations from the cis chromosomal trend, this trend was subtracted from the data and tests performed on the zero-calibrated expression values. After successful calibration, the magenta trend line traces the *x*-axis; and significant deviations from the trend are shown as blue

dots [low | medium | high resolution]. We here also show the 100 genes most strongly deviating from the cis trend in the context of an *M(A)* plot and their chromosomal locations [chr5to0.MA | chr5to0.Mpos]. Also when all genes significantly differentially expressed relatively to the chromosomal cis-trend are included in the figure [chr5to0.Mpos.plain], effects appear randomly distributed over the chromosome. Rainbow colours indicate relative significance (red/yellow is highest, blue/magenta is lowest). Only a minority of genes is below the general cis-trend and therefore dosage compensated or down-regulated.
Figure S3 highlights in red the six epigenetic modifiers located on the triplicated chromosome that are discussed in our manuscript (see legend). All six fully follow the cis chromosomal trend of increased expression. Similarly, as an example of trans effects, **Figure S4** surveys differential regulation on chromosome 2. Red highlights show the two epigenetic modifiers ROS1 and RDR5, prominently up-regulated in the trisomic plants.

Genes Affected – Trends: A discussion of Dosage Compensation

We considered two approaches for studying dosage compensation:

1. For the data shown in Figure 5, we could apply standard tests for differential expression. For the trend estimates in the manuscript, we used two independent statistical tests:
    1. a convex decreasing density estimate for the number of non-Null hypothesis, giving a lower bound of 3%, and
    2. an empirical Bayes regularized *t*-test with Benjamini-Yekutieli adjustment for multiple testing and a False Discovery Rate (FDR) threshold of 5%.

   Raising this threshold did not increase the estimated number of significantly different genes, as the percentage of False Positives expected increases fast with a higher FDR threshold. We can consider genes with an expression level that is significantly higher in trisomics than in disomics as not fully dosage compensated. Then we can designate genes with no significant change in expression as dosage compensated genes. With the FDR controlled at 5%, the actual percentage of False Positives can be anywhere between 0% and 5%. Test results therefore gave an upper bound of 15% for the 'best case' of an FDR of 0%, and 11% for the 'worst case' of an FDR of 5%. This analysis approach has the advantage that we can also discern 'overshooting' dosage compensation:- about 1% of genes in trisomic samples were actually more weakly expressed. The disadvantage of this method is that it does not consider partly dosage compensated genes.
2. A complementary approach therefore considers the deviation from the chromosome 5 trend. This fully considers partial dosage compensation. Under ideal measurement conditions, this would correspond to identifying genes with a significant deviation from 1.5× change. In realistic conditions, this test can be performed by subtracting the average chromosome 5 trend from the data, yielding a zero-centred distribution [cal0M]. We can then consider genes on chromosome 5 with an expression level that is significantly lower than the chromosome trend to be dosage compensated. This is again assessed by an empirical Bayes regularized *t*-test, Benjamini-Yekutieli adjustment for multiple testing, and an FDR

threshold of 5%. Raising this threshold does not increase the estimated number of significantly different genes, as the percentage of False Positives expected increases with a higher FDR threshold. This gives an independent estimate of 14% of (somewhat) dosage compensated genes (and with a 5% FDR, the lower bound is 13%). Intuitively, how much gene expression needs to be lower to be considered significant is determined by the standard deviation of the random scatter of gene expression on the chromosome.

In summary, at least 13% of genes were dosage compensated, at least 3% of genes were fully dosage compensated, whereas at least 85% of genes were not dosage compensated. The manuscript text collects these summaries in a concise form, with test details given in the Methods section. Evidence from individual tests is quoted in parentheses in the main manuscript text.

Genes Affected – Annotated Tables

These tables are TAB-delimited text files that can be loaded into any spreadsheet program. The largest tables are about 20MB in size. Subsets considering only strongly expressed genes are marked as *bright*. Calibrated cis data tested for deviation from the chromosomal trend is labelled *Chr5to0*. [***Tables in Online Supplement only***]

GOslim trends

The tables below are small TAB-delimited text files and can be loaded into any spreadsheet program or text editor. The first column shows the odds-ratio (OR) for the GOslim group being overrepresented in the test-set *vs* the entire chip, then follows a Holm adjusted FWER *p*-value, the relative (percentage) and absolute counts in test and reference sets, and the GOslim group being tested. Groups are sorted by significance of over- or underrepresentation in the test set. Only strongly expressed (*bright*) genes were considered, as our assay is most accurate for these.
[***Tables in Online Supplement only***]

Supporting Material:

Text of the Online Supplement archived at

http://bioinf.boku.ac.at/pub/trisomy2008

For convenience, these pages also include the text of the Online Supplement albeit without figures, whereas the Online Supplement provides many complementary figures and tables fully linked from the online text.

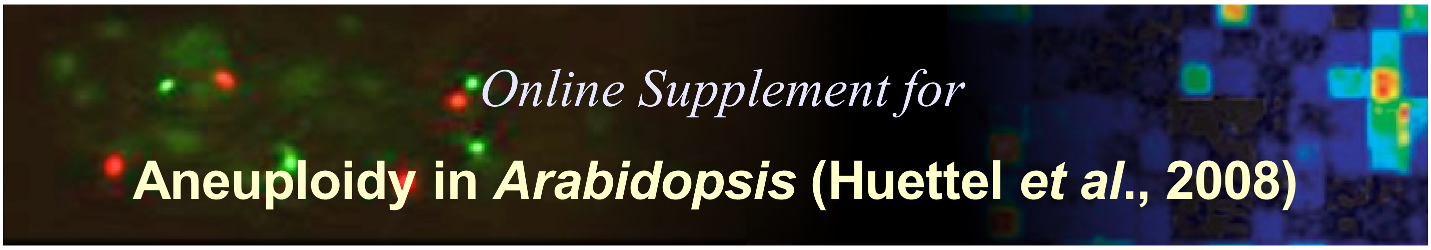

Effects of Aneuploidy on Genome Structure, Expression and Interphase Organization in *Arabidopsis thaliana*

Bruno Huettel,[1] David P. Kreil,[1] Marjori Matzke,[*] and Antonius J. M. Matzke
